# Causal associations of sarcopenia‐related traits with cardiometabolic disease and Alzheimer's disease and the mediating role of insulin resistance: A Mendelian randomization study

**DOI:** 10.1111/acel.13923

**Published:** 2023-07-05

**Authors:** Chaojie Ye, Lijie Kong, Yiying Wang, Jie Zheng, Min Xu, Yu Xu, Mian Li, Zhiyun Zhao, Jieli Lu, Yuhong Chen, Weiqing Wang, Guang Ning, Yufang Bi, Tiange Wang

**Affiliations:** ^1^ Department of Endocrine and Metabolic Diseases Shanghai Institute of Endocrine and Metabolic Diseases, Ruijin Hospital, Shanghai Jiao Tong University School of Medicine Shanghai China; ^2^ Shanghai National Clinical Research Center for Metabolic Diseases, Key Laboratory for Endocrine and Metabolic Diseases of the National Health Commission of the PR China, Shanghai Key Laboratory for Endocrine Tumor, Ruijin Hospital Shanghai Jiao Tong University School of Medicine Shanghai China

**Keywords:** Alzheimer's disease, cardiometabolic disease, insulin resistance, Mendelian randomization, sarcopenia

## Abstract

The causal influence of sarcopenia on cardiometabolic disease and Alzheimer's disease and whether and to what extent insulin resistance plays a mediating role therein were unclear. We performed two‐step, two‐sample Mendelian randomization applying genetic instruments of sarcopenia‐related traits based on GWASs from the UK Biobank (up to 461,026 European participants) to examine their causal associations with six cardiometabolic diseases and Alzheimer's disease extracted from large‐scale European descent GWASs with adjustment for body fat percentage and physical activity, and to assess proportions of the causal effects mediated by insulin resistance. Genetic instruments of insulin resistance were derived from the GWASs by Meta‐Analyses of Glucose and Insulin‐related traits Consortium and Global Lipids Genetics Consortium. Each 1‐SD lower grip strength, appendicular lean mass (ALM) and whole‐body lean mass (WBLM), as well as lower walking pace, were causally associated with higher risks of diabetes (odds ratio [OR] range: 1.20 [95% confidence interval: 1.10–1.32] for ALM to 2.30 [1.14–4.68] for walking pace), nonalcoholic fatty liver disease ([NAFLD], 1.33 [1.08–1.64] for ALM to 2.30 [1.02–5.18] for grip strength), hypertension (1.12 [1.05–1.20] for ALM to 4.43 [2.68–7.33] for walking pace), coronary heart disease ([CHD], 1.20 [1.13–1.27] for ALM to 2.73 [1.84–4.05] for walking pace), myocardial infarction ([MI], 1.18 [1.11–1.25] for ALM to 2.47 [1.63–3.73] for walking pace), small vessel stroke (1.25 [1.15–1.37] for ALM to 1.29 [1.10–1.52] for WBLM), and Alzheimer's disease (1.10 [1.05–1.15] for ALM to 1.28 [1.19–1.38] for WBLM). These causal associations were largely independent of body fat percentage and physical activity. Insulin resistance mediated 16%–34% of the effect of grip strength and 7%–28% of the effect of ALM on diabetes, NAFLD, hypertension, CHD, and MI. The direct effect of WBLM on diabetes diminished toward null with adjustment for insulin resistance. We found no evidence that insulin resistance was on the causal pathways from walking pace to the studied disease outcomes. Causal findings from the inverse‐variance weighted method were validated by sensitivity analyses. These findings support improving sarcopenia‐related traits as precautions against major cardiometabolic diseases and Alzheimer's disease, with particular emphasis on insulin resistance as a target in the intervention of sarcopenia‐related cardiometabolic risk.

## Graphical abstract

This study shed light on the causal influences of sarcopenia‐related traits on major cardiometabolic diseases and Alzheimer's disease independent of body fat and physical activity and the mediating role of insulin resistance in the association pathway. These findings support improving sarcopenia as encouraging precautions against major cardiometabolic diseases and Alzheimer's disease, with a particular emphasis on insulin resistance as a target in lowering sarcopenia‐related cardiometabolic risk.

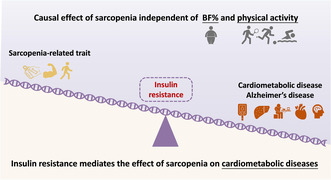

## 1 INTRODUCTION

Sarcopenia is generally defined as an age‐related progressive and generalized decline in skeletal muscle mass and function that dramatically affects health status and life quality (Cruz‐Jentoft & Sayer, 2019). Emerging epidemiological studies have indicated possible relationships of sarcopenia‐related traits with multiple cardiometabolic diseases (e.g., type 2 diabetes, nonalcoholic fatty liver disease [NAFLD], and cardiovascular disease [CVD]) and Alzheimer's disease (Beeri et al., 2021; Bhanji et al., 2017; Gao et al., 2022; Petermann‐Rocha et al., 2022; Scott et al., 2016), suggesting sarcopenia to be a promising investment to augment the early prevention of these age‐related diseases.

However, uncertainty remains concerning the causal influence of sarcopenia on health outcomes and the potential mediating pathway, which limits the translation of current knowledge about the pathophysiology of sarcopenia into clinical practice (Haase et al., 2022). First, current evidence for the associations of sarcopenia with cardiometabolic diseases and Alzheimer's disease is largely derived from observational studies (Beeri et al., 2021; Bhanji et al., 2017; Cruz‐Jentoft & Sayer, 2019; Gao et al., 2022; Petermann‐Rocha et al., 2022; Scott et al., 2016). Evidence from Mendelian randomization studies was limited to cardiometabolic diseases (Chen et al., 2023; Park et al., 2022; Zhao et al., 2022), and it is unknown whether these associations are independent of excessive body fat and physical inactivity, which are typically related to sarcopenia (Batsis & Villareal, 2018) (Yuan & Larsson, 2023). Investigating the causal effect of sarcopenia on cardiometabolic diseases and Alzheimer's disease accounting for the effect of body fat and physical activity is critical to dissecting the independent effect of sarcopenia and identifying individuals at high risk of these diseases. Second, insulin resistance is one of the major pathophysiological mechanisms in the development of cardiometabolic disease and Alzheimer's disease (Chalasani et al., 2012; Sędzikowska & Szablewski, 2021; Wang et al., 2020; Wang et al., 2022). Because skeletal muscle is the primary tissue responsible for insulin‐regulated glucose metabolism, low skeletal muscle mass may reduce insulin‐mediated glucose disposal, which combined with increased insulin resistance during skeletal muscle ageing, is involved in the cascades that induce disease progression (DeFronzo & Tripathy, 2009). Therefore, investigating the mediating role of insulin resistance in the associations of sarcopenia with cardiometabolic diseases and Alzheimer's disease could enhance the understanding of the pathogenesis from sarcopenia to the diseases and expose potential targets for diminishing sarcopenia‐related disease risks. Few studies have investigated the mediating pathway from sarcopenia to these health outcomes thus far.

To fill the knowledge gap, we aimed to perform a Mendelian randomization (MR) study applying genetic instruments to proxy for sarcopenia‐related traits to assess the causal, independent associations of sarcopenia‐related traits with six major cardiometabolic diseases and Alzheimer's disease, with particular interests in evaluating whether and to what extent insulin resistance plays a mediating role in these associations.

## 2 METHODS

### 2.1 Study design

This MR study consisted of two analysis phases. In phase 1, we first examined the causal effects of four sarcopenia‐related traits on six cardiometabolic diseases and Alzheimer's disease, and then investigated whether the causal effects were independent of body fat percentage (BF%) and physical activity. In phase 2, we assessed the mediating role of insulin resistance in the causal associations between sarcopenia‐related traits and these disease outcomes. We conducted MR analyses under three fundamental assumptions: (1) the single nucleotide polymorphisms (SNPs) are associated with the exposure of interest (the relevance assumption); (2) the SNPs share no common cause with the outcome (the independence assumption); and (3) the SNPs do not affect the outcome except through the exposure (the exclusion restriction assumption) (Davies et al., 2018). Due to the fixed nature of the genotype from its random formation at conception following Mendel's First and Second Laws of Inheritance, MR results are less likely to be influenced by reverse causation and confounding (Davies et al., 2018). We reported this study according to the Strengthening the Reporting of Observational Studies in Epidemiology using Mendelian Randomization (STROBE‐MR) (Skrivankova et al., 2021). This study was conducted using the genome‐wide association study (GWAS) summary‐level data in predominantly European individuals from reliable consortia or studies. Information on GWAS datasets used in this study is shown in Table 1. Ethical approval for the GWASs can be found in the cited GWAS publications below.

**TABLE 1:** Information of GWAS datasets used in the MR study.

Phenotype	Unit	Sample size (case/control)	Population	Consortium or cohort study	Year of publication	PMID
Exposure: sarcopenia‐related trait						
Grip strength	1‐SD	461,026	European	UK Biobank	2018	29846171
ALM	1‐SD	450,243	European	UK Biobank	2020	33097823
WBLM	1‐SD	454,850	European	UK Biobank	2018	29846171
Walking pace	A shift of one category (brisk, steady/average, slow)	450,967	European	UK Biobank	2020	33128006
Covariate: body fat and physical activity						
BF%	1‐SD	454,633	European	UK Biobank	2018	29846171
Physical activity (device‐measured)	1‐SD	91,084	European	UK Biobank	2018	29899525
Mediator: insulin resistance trait						
Insulin resistance (fasting insulin)	Natural log‐transformed pmol/L	151,013	European	MAGIC	2021	34059833
Insulin resistance phenotype^a^	1‐SD	188,577	European	MAGIC, GLGC	2017	27841877, 29046328
Outcome: cardiometabolic disease and Alzheimer's disease						
Type 2 diabetes	Event	12,931/57,196	European	Meta	2018	29358691
NAFLD	Event	894/217,898	European	FinnGen Biobank	2021	29846171
Hypertension	Event	42,857/162,837	European	FinnGen Biobank	2021	29846171
CHD	Event	60,801/123,504	77% European	CARDIoGRAMplusC4D	2015	26343387
MI	Event	43,676/128,199	77% European	CARDIoGRAMplusC4D	2015	26343387
Small vessel stroke	Event	5386/192,662	European	ISGC MEGASTROKE	2018	29531354
Alzheimer's disease	Event	85,934/401,577	European	EADB	2022	35379992

Abbreviations: ALM, appendicular lean mass; BF%, body fat percentage; CARDIoGRAMplusC4D, Coronary ARtery Disease Genome wide Replication and Meta‐analysis plus The Coronary Artery Disease Genetics; CHD, coronary heart disease; EADB, European Alzheimer & Dementia Biobank; GLGC, Global Lipids Genetics Consortium; GWAS, genome‐wide association study; HDL‐C, high‐density lipoprotein cholesterol; ISGC, International Stroke Genetics Consortium; MAGIC, Meta‐Analyses of Glucose and Insulin‐related traits Consortium; MI, myocardial infarction; MR, Mendelian randomization; NAFLD, nonalcoholic fatty liver disease; SD, standard deviation; WBLM, whole‐body lean mass.

^a^ Each 1‐SD higher insulin resistance phenotype is equivalent to 55% higher geometric mean of fasting insulin, 0.89 mmol/L higher triglycerides, and 0.46 mmol/L lower HDL‐C.

### 2.2 Data source for and selection of genetic instruments

We selected grip strength and walking pace as measures of muscle function and appendicular lean mass (ALM) and whole‐body lean mass (WBLM) as measures of muscle mass, which are recognized as key components of sarcopenia (Gao et al., 2022; Pei et al., 2020). Genetic instruments for grip strength (*n* = 461,026), ALM (*n* = 450,243), WBLM (*n* = 454,850), and walking pace (*n* = 450,967) were from the largest GWASs from the UK Biobank of participants aged between 48 and 73 years at recruitment (Elsworth et al., 2017; Pei et al., 2020; Timmins et al., 2020). Grip strength was obtained using a Jamar J00105 hydraulic hand dynamometer that can be adjusted for hand size, and WBLM was measured by bioelectrical impedance analysis (BIA) using a Tanita BC418MA body composition analyzer (Elsworth et al., 2017). ALM was quantified by the sum of fat‐free mass at the arms and legs measured by BIA following the same protocol as WBLM (Elsworth et al., 2017). Walking pace was defined as a category phenotype, with self‐reported responses of “slow,” “steady/average,” and “brisk” coded as 0, 1, and 2, respectively (Timmins et al., 2020). Genetic instruments for BF% was based on GWAS data among 454,633 European individuals from the UK Biobank (Elsworth et al., 2017). GWAS data for physical activity were based on the objective accelerometer‐based activity among 91,084 individuals from the UK Biobank, specifically overall mean acceleration in milligravities across at least 72 h of wrist‐worn accelerometer wear (Klimentidis et al., 2018).

As genetic instruments for insulin resistance, we used the 53 variants identified in an integrative GWAS of 188,577 European individuals to associate with the insulin resistance phenotype (higher fasting insulin levels adjusted for body mass index [BMI], higher triglyceride levels, and lower high‐density lipoprotein cholesterol [HDL‐C] levels) (Lotta et al., 2017). Because this original GWAS did not publish β estimates or standard errors (SEs) for the association of each SNP with the insulin resistance phenotype (Lotta et al., 2017), these were obtained from a subsequent MR study (Wang et al., 2017), which followed a well‐established method by meta‐analyzing the absolute value of the standardized β coefficient for each of the 53 variants associated with the three components of the insulin resistance phenotype using a fixed‐effect inverse‐variance weighted (IVW) method to obtain the genetic instruments for the insulin resistance phenotype. The insulin resistance phenotype was equivalent to 55% higher geometric mean of fasting insulin, 0.89 mmol/L higher triglycerides, and 0.46 mmol/L lower HDL‐C (Wang et al., 2017).

In UVMR, we further weighted the 53 variants based on their effects on fasting insulin levels in the latest GWAS meta‐analysis of 151,013 diabetes‐free European individuals conducted by the Meta‐Analyses of Glucose and Insulin‐related traits Consortium (MAGIC) as IVs for insulin resistance (Chen et al., 2021). Besides, since the summary‐level GWAS data for insulin resistance phenotype were not currently available, we maintained the use of the 53 IVs for insulin resistance phenotype and applied all the IV SNPs based on fasting insulin in multivariable MR (MVMR) analyses in the present study.

We extracted genetic variants associated with sarcopenia‐related traits, BF%, and insulin resistance traits at genome‐wide significance (*p* < 5.00 × 10^−8^), and ensured that genetic variants were independent of each other (linkage disequilibrium [LD] *r* ^2^ < 0.001 within 10,000 kb) by using the 1000 Genomes European reference panel16 and PLINK v1.917.

### 2.3 Data source for cardiometabolic disease and Alzheimer's disease

Cardiometabolic diseases included type 2 diabetes, NAFLD, hypertension, coronary heart disease (CHD), myocardial infarction (MI), and small vessel stroke. In order to avoid sample overlap with the exposure GWASs from the UK Biobank, GWAS summary statistics for diabetes were from a study conducting a reanalysis of publicly available diabetes GWASs (12,931 cases and 57,196 controls) (Bonàs‐Guarch et al., 2018). GWAS data for NAFLD (894 cases and 217,898 controls) and hypertension (42,857 cases and 162,837 controls) were from the FinnGen Biobank (Kurki et al., 2023). GWAS data for CHD (60,801 cases and 123,504 controls) and MI (43,676 cases and 128,199 controls) were from the Coronary ARtery Disease Genome wide Replication and Meta‐analysis plus The Coronary Artery Disease Genetics (CARDIoGRAMplusC4D) consortium (Nikpay et al., 2015). GWAS data for small vessel stroke (stroke caused by small vessel disease) were from the MEGASTROKE consortium (Malik et al., 2018), in which analysis for small vessel stroke in European individuals involved 5386 stroke cases and 192,662 controls. GWAS data for Alzheimer's disease were from the latest GWAS by the European Alzheimer & Dementia Biobank (EADB) based on 85,934 cases and 401,577 controls (Bellenguez et al., 2022).

### 2.4 Statistical analysis

We presented MR estimates as odds ratios (ORs) with 95% confidence intervals (CIs) for binary outcomes and β coefficients with SEs for continuous outcomes. We assessed the causal associations of grip strength, ALM, WBLM, and walking pace with six cardiometabolic diseases and Alzheimer's disease by applying univariable MR (UVMR), with estimates denoted as β0. Then, we applied multivariable MR (MVMR) to assess the causal effect of grip strength, ALM, WBLM, and walking pace on the diseases with adjustment for BF% and physical activity to determine whether sarcopenia‐related traits were causally associated with the outcomes independent of BF% and physical activity.

We performed two‐step MR to explore whether insulin resistance has a mediation effect on the causal associations between sarcopenia‐related traits and the outcomes. The first step was to use UVMR to estimate the causal effect of each sarcopenia‐related trait on insulin resistance, with each estimate denoted as β1. Meanwhile, reverse causation between insulin resistance and each sarcopenia‐related trait was tested using UVMR to ensure that no bidirectionality might affect the validity of the mediation model. The second step was to estimate the causal effect of insulin resistance on each outcome using UVMR and MVMR with adjustment for each sarcopenia‐related trait and to denote the MVMR estimate as β2. The mediation proportion of insulin resistance in the causal association between each sarcopenia‐related trait and outcome was calculated as the product of β1 and β2 divided by β0 (Carter et al., 2021), and the 95% CI of the mediation proportion was obtained using the delta method (MacKinnon et al., 2002).

The IVW method was used as the main analysis. The *F* statistic was employed to evaluate the issue of weak instrument in MR analysis. Cochran's Q statistic was calculated to assess the heterogeneity of the instrumental variables (IVs), and the main analyses were performed using the random‐effect IVW method that combines Wald ratios (or Ratio estimates) together in an IVW meta‐analysis adjusting for heterogeneity. Several sensitivity analyses were conducted to assess the robustness of the IVW results. For UVMR, we performed four sensitivity analyses including the weighted median, the weighted mode, the MR Egger, and the MR pleiotropy residual sum and outlier (PRESSO) methods. The weighted median method can provide consistent estimates as long as at least 50% of the information contributing to the analysis comes from valid IVs (Bowden et al., 2016). The weighted mode method produces robust estimates when the largest number of similar individual‐instrument causal effect estimates arise from valid IVs, even if most are invalid (Bowden et al., 2016). The MR Egger method can detect and adjust for the bias due to directional pleiotropy under the InSIDE (Instrument Strength Independent of Direct Effect) assumption (Bowden et al., 2016). The MR PRESSO method detects outlying SNPs that are potentially horizontally pleiotropic and evaluates whether exclusion of outlying SNPs influences the causal estimates (Verbanck et al., 2018). For MVMR, multivariable MR Egger (MVMR Egger) method was performed as sensitivity analysis. Where SNPs for the exposure were not available in summary statistics of longevity outcomes, we replaced them with proxy SNPs in high LD (*r* ^2^ > 0.8) identified on the online platform LDlink (https://ldlink.nci.nih.gov/).

All MR analyses were performed using R packages *TwoSampleMR*, *MVMR*, and *MRPRESSO* in R software (version 4.0.3; R Development Core Team, Vienna, Austria). The statistical significance level was set to be 0.05. In the analyses for the causal associations of four sarcopenia‐related traits and BF% with cardiometabolic disease and Alzheimer's disease, the false discovery rate (FDR) q value was calculated by the Benjamini–Hochberg method to address multiple hypothesis testing (Glickman et al., 2014). Significant results in both IVW and at least one sensitivity analysis in UVMR or significant results in both multivariable IVW (MV‐IVW) and MVMR Egger analyses in MVMR indicate causal associations.

## 3 RESULTS

### 3.1 Unadjusted and BF%‐ and physical activity‐adjusted causal effects of sarcopenia‐related traits on cardiometabolic disease and Alzheimer's disease

**FIGURE 1:**
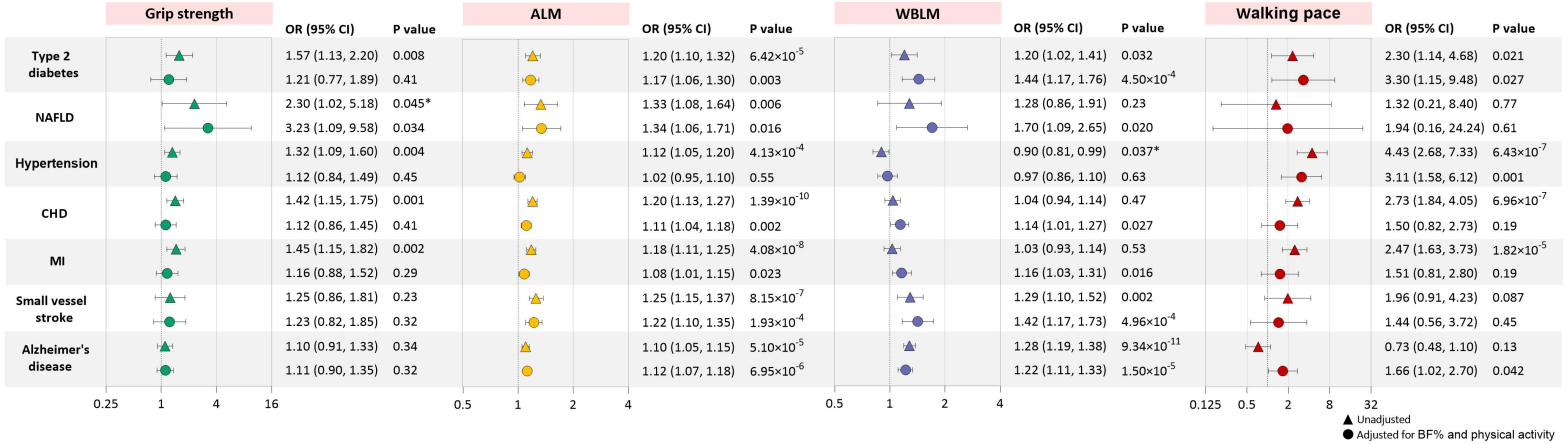
Unadjusted and BF%‐ and physical activity‐adjusted causal effects of sarcopenia‐related traits on cardiometabolic diseases and Alzheimer's disease. ORs (95% CIs) represent risks for cardiometabolic diseases and Alzheimer's disease associated with each 1‐SD lower grip strength, ALM, WBLM, and a shift of one category to a lower walking pace (brisk, steady/average, and slow). *The unadjusted causal effect of grip strength on NAFLD (FDR q value = 0.061) and WBLM on hypertension (FDR q value = 0.052) turned borderline significant after FDR‐adjustment for multiple comparisons. ALM, appendicular lean mass; BF%, body fat percentage; CHD, coronary heart disease; CI, confidence interval; FDR, false discovery rate; MI, myocardial infarction; NAFLD, nonalcoholic fatty liver disease; OR, odds ratio; SD, standard deviation; WBLM, whole‐body lean mass.

In the IVW method, each 1‐standard deviation (SD) lower genetically determined grip strength was associated with increased risks of type 2 diabetes (OR: 1.57; 95% CI: 1.13–2.20), NAFLD (2.30; 1.02–5.18), hypertension (1.32; 1.09–1.60), CHD (1.42; 1.15–1.75), and MI (1.45; 1.15–1.82), but not small vessel stroke (1.25; 0.86–1.81) and Alzheimer's disease (1.10; 0.91–1.33; Figure 1, Table S1). Each 1‐SD lower genetically determined ALM was associated with increased risks of all six cardiometabolic diseases and Alzheimer's disease, and the OR (95% CI) ranged from 1.33 (1.08–1.64) for NAFLD to 1.10 (1.05–1.15) for Alzheimer's disease. Each 1‐SD lower genetically determined WBLM was associated with increased risks of diabetes (OR: 1.20; 95% CI: 1.02–1.41), small vessel stroke (1.29; 1.10–1.52), and Alzheimer's disease (1.28; 1.19–1.38) and a decreased risk of hypertension (0.90; 0.81–0.99). Genetically determined lower walking pace was causally associated with increased risks of diabetes (OR: 2.30; 95% CI: 1.14–4.68), hypertension (4.43; 2.68–7.33), CHD (2.73; 1.84–4.05), and MI (2.47; 1.63–3.73). The causal effects of grip strength on NAFLD (FDR q value = 0.061) and WBLM on hypertension (FDR q value = 0.052) turned borderline significant after FDR‐adjustment for multiple comparisons.

Although the MR Egger pleiotropy test indicated potential horizontal pleiotropy between grip strength and hypertension (Table S2), the causal association between grip strength and hypertension still existed (PRESSO‐estimated OR associated with each 1‐SD lower grip strength: 1.37; 95% CI: 1.14–1.65) after excluding one outlying SNP detected by the MR PRESSO method (Table S1). Estimates for Cochran's Q statistic indicated that heterogeneity from genetic instruments might exist.

Genetically determined higher BF% was causally associated with increased risks of diabetes, NAFLD, hypertension, CHD, and MI and a decreased risk of Alzheimer's disease, but not small vessel stroke (Table S3). After adjusting for BF% and physical activity, the causal associations remained for at least one sarcopenia‐related trait with all cardiometabolic diseases and Alzheimer's disease (Figure 1, Table S4).

### 3.2 Causal effects of sarcopenia‐related traits on insulin resistance

In UVMR, each 1‐SD genetically determined loss of grip strength, ALM, and WBLM were associated with higher levels of insulin resistance (IVW‐estimated β for log‐transformed pmol/L fasting insulin: 0.064, 95% CI: 0.016–0.113 for grip strength; 0.027, 0.011–0.042 for ALM; 0.081, 0.056–0.106 for WBLM; Table S5), but not walking pace. While in reverse MR analyses, no causal effect of insulin resistance (fasting insulin) on grip strength, ALM, WBLM, or walking pace was observed.

The MR Egger pleiotropy test indicated potential horizontal pleiotropy between ALM and insulin resistance (fasting insulin), but the MR PRESSO method provided consistent results with the IVW method after the exclusion of 28 potentially pleiotropic SNPs (Tables S5 and S6). There might exist heterogeneity among IVs.

### 3.3 Causal effect of insulin resistance on cardiometabolic disease and Alzheimer's disease

In UVMR, genetically determined insulin resistance (1‐log increment in pmol/L fasting insulin) was associated with increased risks of all six cardiometabolic diseases, with ORs (95% CIs) ranging from 23.98 (10.88–52.86) for diabetes to 2.35 (1.24–4.45) for small vessel stroke, but was not associated with Alzheimer's disease (Table 2, Table S7). Genetically determined each 1‐SD higher insulin resistance phenotype showed consistently causal associations with six cardiometabolic diseases, and ORs (95% CIs) ranged from 4.24 (2.92–6.16) for diabetes to 1.60 (1.20–2.15) for small vessel stroke.

**TABLE 2:** UVMR estimates for the causal associations of insulin resistance with cardiometabolic diseases and Alzheimer's disease.

Insulin resistance trait	Outcome	OR (95% CI)^a^	*p* value
Insulin resistance (fasting insulin)	Type 2 diabetes	23.98 (10.88, 52.86)	3.32 × 10^−15^
	NAFLD	17.52 (3.30, 92.93)	7.70 × 10^−4^
	Hypertension	3.12 (2.16, 4.49)	1.09 × 10^−9^
	CHD	2.93 (2.00, 4.30)	3.59 × 10^−8^
	MI	2.54 (1.70, 3.79)	5.62 × 10^−6^
	Small vessel stroke	2.35 (1.24, 4.45)	0.009
	Alzheimer's disease	1.12 (0.86, 1.44)	0.40
Insulin resistance phenotype	Type 2 diabetes	4.24 (2.92, 6.16)	2.68 × 10^−14^
	NAFLD	3.61 (1.57, 8.32)	0.003
	Hypertension	1.60 (1.32, 1.95)	2.21 × 10^−6^
	CHD	1.82 (1.55, 2.14)	2.78 × 10^−13^
	MI	1.79 (1.51, 2.11)	6.29 × 10^−12^
	Small vessel stroke	1.60 (1.20, 2.15)	0.002
	Alzheimer's disease	1.00 (0.89, 1.13)	0.98

Abbreviations: CHD, coronary heart disease; CI, confidence interval; HDL‐C, high‐density lipoprotein cholesterol; MI, myocardial infarction; NAFLD, nonalcoholic fatty liver disease; OR, odds ratio; SD, standard deviation; UVMR, univariable Mendelian randomization.

^a^ ORs (95% CIs) represent risks for cardiometabolic diseases and Alzheimer's disease associated with each 1‐log unit higher insulin resistance (pmol/L fasting insulin) or 1‐SD higher insulin resistance phenotype (equivalent to 55% higher geometric mean of fasting insulin, 0.89 mmol/L higher triglycerides, and 0.46 mmol/L lower HDL‐C).

**FIGURE 2:**
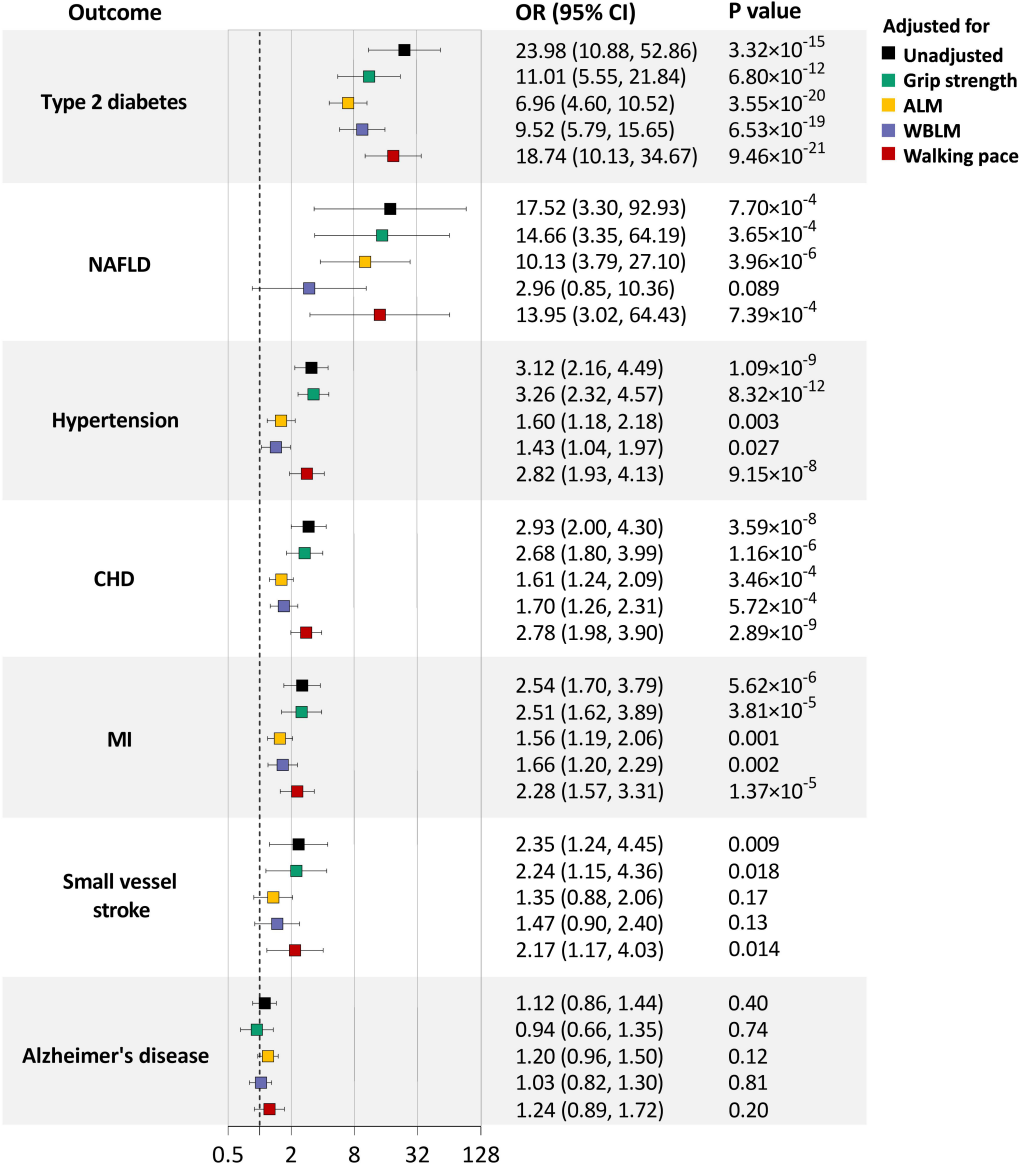
UVMR and MVMR estimates for the causal associations of insulin resistance with cardiometabolic diseases and Alzheimer's disease. ORs (95% CIs) represent risks for cardiometabolic diseases and Alzheimer's disease associated with each 1‐log unit higher insulin resistance (pmol/L fasting insulin). ALM, appendicular lean mass; CHD, coronary heart disease; CI, confidence interval; MI, myocardial infarction; MVMR, multivariable Mendelian randomization; NAFLD, nonalcoholic fatty liver disease; OR, odds ratio; UVMR, univariable Mendelian randomization; WBLM, whole‐body lean mass.

In MVMR, the causal associations of insulin resistance (fasting insulin) with cardiometabolic diseases were not substantially changed with adjustment for grip strength or walking pace, but were diminished with adjustment for ALM or WBLM, especially the associations with NAFLD and small vessel stroke (Figure 2, Table S8).

### 3.4 Mediation effect of insulin resistance in the associations between sarcopenia‐related traits and cardiometabolic diseases

**FIGURE 3:**
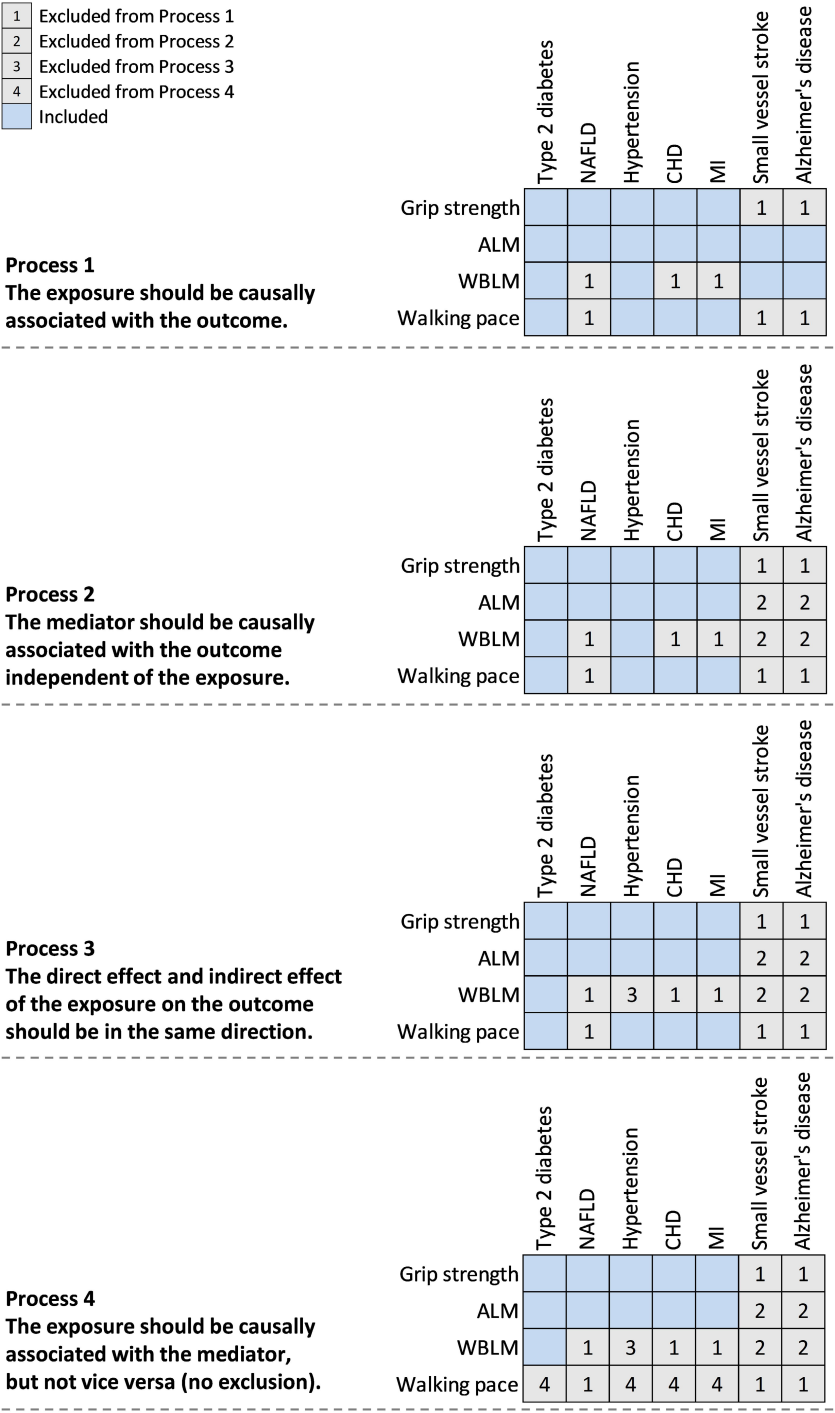
Overview of the process for exploring exposure‐insulin resistance‐outcome causal pathways by two‐step MR. Two‐step MR was performed to explore the mediating role of insulin resistance in the causal associations of sarcopenia‐related traits with cardiometabolic diseases and Alzheimer's disease based on the following processes: (1) the exposure should be causally associated with the outcome; (2) the mediator should be causally associated with the outcome independent of the exposure; (3) the direct effect and indirect effect of the exposure on the outcome should be in the same direction; and (4) the exposure should be causally associated with the mediator, but not vice versa. ALM, appendicular lean mass; CHD, coronary heart disease; MI, myocardial infarction; MR, Mendelian randomization; NAFLD, nonalcoholic fatty liver disease; WBLM, whole‐body lean mass.

**FIGURE 4:**
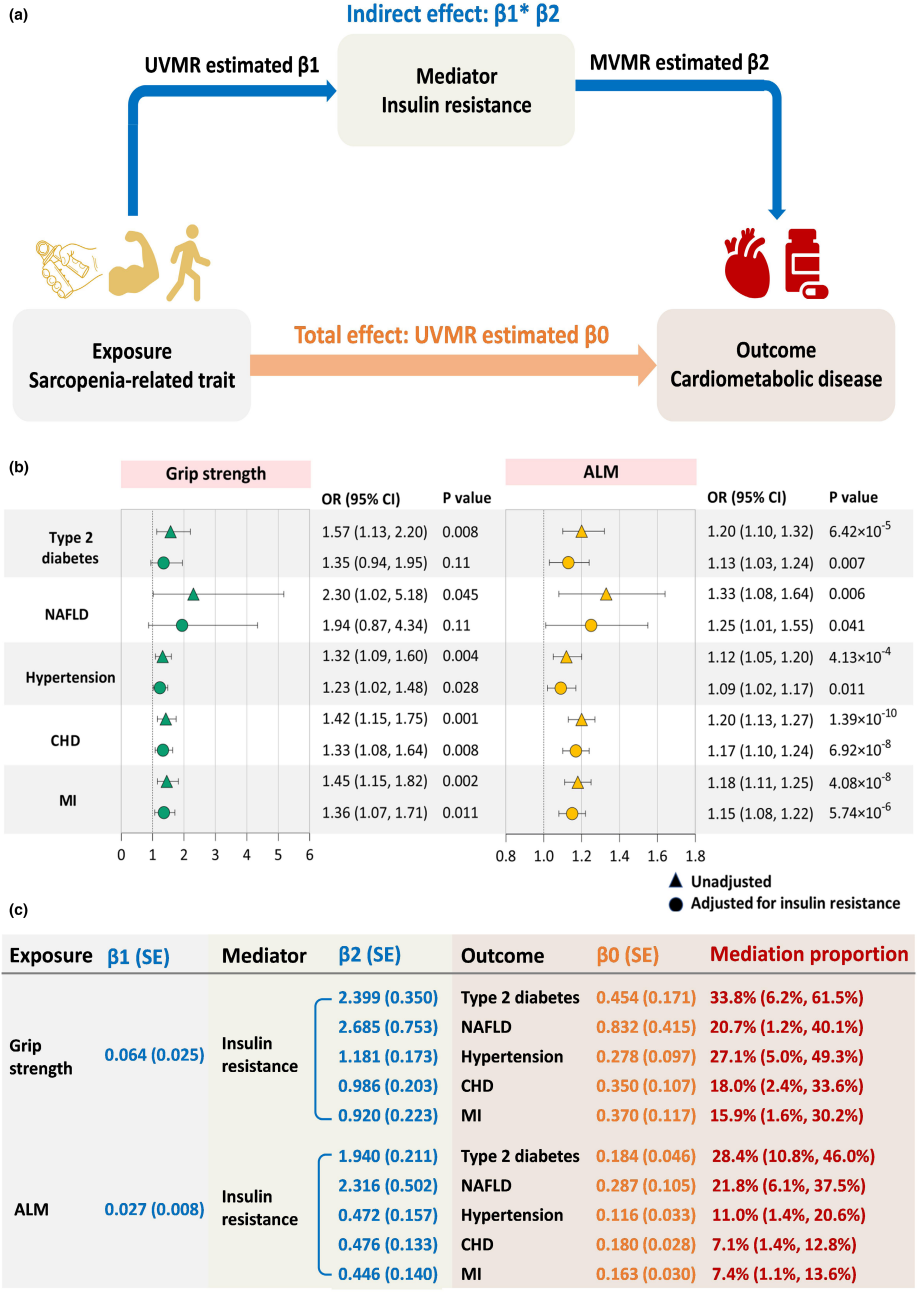
Two‐step MR framework and estimated proportion mediated by insulin resistance in the causal associations between sarcopenia‐related traits and cardiometabolic diseases. (a) Two‐step MR framework. (b) Causal effects of sarcopenia‐related traits on cardiometabolic diseases with adjustment for insulin resistance. (c) Proportion mediated by insulin resistance in the causal associations between sarcopenia‐related traits and cardiometabolic diseases. Two‐step MR was used to evaluate the mediating role of insulin resistance in the causal associations of grip strength and ALM with five cardiometabolic diseases. The first step was to estimate the causal effect of each sarcopenia‐related trait on insulin resistance by UVMR, with each estimate denoted as β1. The second step was to estimate the causal effect of insulin resistance on each outcome using MVMR with adjustment for each sarcopenia‐related trait and to denote the MVMR estimate as β2. The mediation proportion of insulin resistance in the association between each sarcopenia‐related trait and outcome was calculated as the product of β1 and β2 divided by the total causal effect of each sarcopenia‐related trait on the outcome (β0), and the 95% CI of the mediation proportion was obtained using the delta method. ALM, appendicular lean mass; CHD, coronary heart disease; CI, confidence interval; MI, myocardial infarction; MR, Mendelian randomization; MVMR, multivariable Mendelian randomization; NAFLD, nonalcoholic fatty liver disease; OR, odds ratio; SE, standard error; UVMR, univariable Mendelian randomization.

After a series of processes for exploring exposure‐insulin resistance‐outcome causal pathways (Figure 3), we applied the two‐step MR to assess the role of insulin resistance in mediating the causal effects of sarcopenia‐related traits on five cardiometabolic diseases (Figure 4a). The effects of grip strength and ALM on type 2 diabetes, NAFLD, hypertension, CHD, and MI were partly attenuated after adjusting for insulin resistance (fasting insulin) (Figure 4b, Table S9). Insulin resistance explained 33.8% (95% CI: 6.2%–61.5%) of the total effect of grip strength on diabetes, followed by hypertension (27.1%; 5.0%–49.3%), NAFLD (20.7%; 1.2%–40.1%), CHD (18.0%; 2.4%–33.6%), and MI (15.9%; 1.6%–30.2%; Figure 4C). The proportion mediated by insulin resistance in the associations between ALM and outcomes was 28.4% (95% CI: 10.8%–46.0%) for diabetes, 21.8% (6.1%–37.5%) for NAFLD, 11.0% (1.4%–20.6%) for hypertension, 7.4% (1.1%–13.6%) for MI, and 7.1% (1.4%–12.8%) for CHD. The causal association between WBLM and diabetes was eliminated (OR per 1‐SD higher WBLM: 0.99; 95% CI: 0.84–1.17) after adjusting for insulin resistance, suggesting this association was largely affected by insulin resistance (Table S9).

## 4 DISCUSSION

This MR study investigated the independent causal effects of sarcopenia‐related traits, including loss of grip strength, ALM, WBLM, and walking pace, on six cardiometabolic diseases and Alzheimer's disease, and whether these effects occurred through a causal pathway of insulin resistance. We found that genetically determined lower grip strength, ALM, WBLM, and walking pace were causally associated with increased risks of type 2 diabetes, NAFLD, hypertension, CHD, MI, small vessel stroke, and Alzheimer's disease. The causal effects of sarcopenia‐related traits on these cardiometabolic diseases and Alzheimer's disease were independent of BF% and physical activity. In particular, we identified the mediating effect of insulin resistance in the causal associations between sarcopenia‐related traits and five cardiometabolic diseases, with mediation proportions of 16%–34% for the effect of grip strength and 7%–28% for the effect of ALM on diabetes, NAFLD, hypertension, CHD, and MI. The direct effect of WBLM on diabetes was diminished toward null with adjustment for insulin resistance, suggesting that this association was largely attributable to insulin resistance. These findings support an independent causal impact of sarcopenia‐related traits on greater risks of major cardiometabolic diseases and Alzheimer's disease, and illustrate the substantial mediating role of insulin resistance between sarcopenia and cardiometabolic diseases.

The focus on sarcopenia is one of the initiatives that science and societies look for to address the social and health problems exacerbated by the global population ageing (Gao et al., 2022). Guidance from the European Working Group on Sarcopenia in Older People and the International Conference on Frailty and Sarcopenia Research Task Force has recommended that skeletal muscle function and muscle mass are fundamental to a definitive clinical diagnosis of sarcopenia (Cruz‐Jentoft et al., 2019; Vellas et al., 2018). Grip strength is the most robust indicator of muscle function and mass, with emerging epidemiological studies associating a lower grip strength with increased risks of type 2 diabetes (Li et al., 2016; Yeung et al., 2019), NAFLD (Petermann‐Rocha et al., 2022), and CVD (Celis‐Morales et al., 2018; Tikkanen et al., 2018). On the contrary, current evidence regarding the relationship between muscle mass and health outcomes is inconsistent (Farmer et al., 2019; Li et al., 2016; Tyrovolas et al., 2020). Analyses from the MAILES Study and the UK Biobank demonstrated that reduced muscle mass was not associated with increased risks of diabetes and CVD (Farmer et al., 2019; Li et al., 2016). By contrast, the ATTICA study of 1019 participants aged 45 years or older showed that individuals in the highest tertile of skeletal muscle mass measured by ALM standardized by BMI had 81% lower risk for 10‐year CVD risk compared with those in the lowest tertile (Tyrovolas et al., 2020). The conflicting results might be partly due to the reverse causality, residual confounding (e.g., body fat mass), or the differences in muscle mass measurements in these studies.

Taking advantage of genetic variants as robust IVs, our study provided new evidence that genetically predicted lower muscle function and lean mass were strong causal risk factors for diabetes, NAFLD, hypertension, CHD, MI, and small vessel stroke (except for muscle function). Intriguingly, we applied two measures of muscle mass (i.e., ALM and WBLM) in this study and indicated that each 1‐SD lower genetically induced ALM was associated with 12%–33% increased risks of all six investigated cardiometabolic diseases, while WBLM was associated with 20% increased risk of diabetes, 29% increased risk of small vessel stroke, and 10% decreased risk of hypertension. Given that WBLM includes skeletal muscle, smooth muscle, and cardiac muscle, whereas ALM is determined solely by skeletal muscle, it is plausible that ALM has a stronger predictive power for sarcopenia (Pei et al., 2020), and exhibited closer relationships with cardiometabolic outcomes in this study.

In addition, we observed a more independent effect of lean mass indicators compared to muscle function indicators, especially grip strength, on cardiometabolic diseases and Alzheimer's disease with adjustment for BF% and physical activity. A possible explanation is that lean mass and body fat are two distinct body components with different biological significance and different roles in health outcomes (Lee et al., 2018), whereas higher BF% has been reported to be associated with lower grip strength (Pinto Pereira et al., 2022).

More importantly, our study demonstrated the mediating role of insulin resistance and quantified the proportions mediated by insulin resistance between sarcopenia‐related traits and major cardiometabolic diseases. Although the exact mechanism underlying the relationships between sarcopenia and cardiometabolic disease remains unclear, current experimental and epidemiological evidence suggests that insulin resistance may play an essential role in mediation pathways (Chalasani et al., 2012; DeFronzo & Tripathy, 2009; Laakso & Kuusisto, 2014; Wang et al., 2020; Wang et al., 2022). Skeletal muscle accounts for approximately 80% of insulin‐mediated glucose uptake, and thus the loss of skeletal muscle mass and function related to sarcopenia is the primary defect that directly prompts impaired muscle glycogen synthesis and insulin resistance (DeFronzo & Tripathy, 2009). Moreover, insulin regulates glucose metabolism in peripheral tissues (e.g., skeletal muscle and adipose tissue) and modulates vascular function through effects on vasoreactivity, lipid metabolism, and inflammation (DeFronzo & Tripathy, 2009; Laakso & Kuusisto, 2014). Our study identified relatively higher mediation proportions by insulin resistance between sarcopenia and diabetes and NAFLD compared with hypertension and CVD subtypes, which is in line with previous evidence that insulin resistance per se is a vital early manifestation of diabetes and NAFLD (Chalasani et al., 2012; DeFronzo & Tripathy, 2009), but it may be an initiator that triggers the downstream mechanisms leading to hypertension and CVDs (Laakso & Kuusisto, 2014).

Notably, our MR results established a causal relationship of muscle mass but not muscle function with Alzheimer's disease, which is partially different from current evidence (Beeri et al., 2021). Furthermore, in our MR study, based on the latest and largest GWASs of insulin resistance and Alzheimer's disease, insulin resistance was not associated with Alzheimer's disease and therefore might not mediate the pathway between sarcopenia and Alzheimer's disease. Emerging evidence indicates that insulin dysregulation could influence the clearance of the amyloid β peptide and phosphorylation of tau, which are hallmarks of Alzheimer's disease (Kellar & Craft, 2020). However, sarcopenia characterized by loss of skeletal muscle mass and function mainly leads to peripheral insulin resistance, while Alzheimer's disease is more likely to be a neurodegeneration disorder due to brain insulin resistance (Kellar & Craft, 2020). The above evidence could, to some extent, explain our findings, but the precise mechanism warrants future investigation.

To the best of our knowledge, this study for the first time provided reliable causal evidence for the adverse impact of sarcopenia on major cardiometabolic diseases and Alzheimer's disease independent of BF% and physical activity, and elucidated a potential pathway mediated by insulin resistance between sarcopenia and cardiometabolic diseases. A recent randomized controlled trial has explored a plausible multicomponent intervention that comprised a combination of moderate‐intensity physical activity and nutritional counseling for sarcopenia (Bernabei et al., 2022). Insulin resistance improvements such as lifestyle intervention and medication therapy also offer opportunities for reducing the burden of cardiometabolic diseases attributable to sarcopenia (Wang et al., 2020; Wang et al., 2022). Our findings add to the understanding of the pathophysiology of cardiometabolic diseases and inform the development of physical and pharmacotherapeutic interventions targeting sarcopenia or its causative pathway, such as insulin resistance, to diminish the related disease burden.

The strengths of this study included the rigorously designed MR analytical framework, the comprehensive measures of sarcopenia using both muscle strength and muscle mass, and the robust findings strengthened by extensive sensitivity analyses. Limitations should also be noted. First, although the concordance of the results from multiple sensitivity analyses and complementary tests indicated that weak IV, horizontal pleiotropy, and outliers that might violate the basic MR assumptions did not significantly influence our causal estimates, the causal associations should be interpreted with caution as several assumptions of the methods are untestable, and heterogeneity of the IVs and residual horizontal pleiotropy might still potentially bias some results. Second, since the summary‐level GWAS data for insulin resistance phenotype were not currently available, we maintained the use of the 53 IVs for insulin resistance phenotype and applied all the IV SNPs based on fasting insulin as a surrogate in MVMR, which might not capture full information on the mediating role of insulin resistance in the causal pathway from sarcopenia‐related traits to cardiometabolic diseases. Nevertheless, we maximized the use of publicly available genetic data for insulin resistance and generated novel causal evidence for the associations between sarcopenia, insulin resistance, and related diseases. Future GWASs identifying SNPs for more direct insulin resistance indicators (e.g., Homeostatic Model Assessment of Insulin Resistance) could facilitate further validation of our current use of the insulin resistance phenotype and the method to generate the β estimates, which would offer valuable insights and enhance the understanding of the causal associations between sarcopenia, insulin resistance, and related diseases. Third, after the FDR‐adjustment for multiple testing, the causal effects of grip strength on NAFLD and WBLM on hypertension turned borderline significant, which may require additional validations with distinct cohorts in future analyses to ensure the robustness and generalizability of these findings. Fourth, our study indicated that insulin resistance mediated part of the effect of sarcopenia on cardiometabolic diseases and did not serve as a mediator between sarcopenia and Alzheimer's disease, leaving the remaining influence of sarcopenia unexplained, which warranted further research. Fifth, the present study was based on GWAS datasets of European descendants, and thus caution should be exercised when generalizing our findings to other ethnic populations.

## 5 CONCLUSIONS

In summary, this study shed light on the causal influence of sarcopenia on major cardiometabolic diseases and Alzheimer's disease independent of body fat and physical activity and the mediating role of insulin resistance in the association pathway. Our findings suggest that improving muscle function and muscle mass is an encouraging precaution against cardiometabolic diseases and Alzheimer's disease, with particular emphasis on insulin resistance as an essential target in the prevention and intervention of sarcopenia‐related cardiometabolic risk. Future analyses with more direct measurements for insulin resistance and with different research strategies are warranted to replicate the findings of this study.

## AUTHOR CONTRIBUTIONS

CY and TW contributed to the conception and design of the study. CY performed statistical analysis and drafted the manuscript. TW critically revised the manuscript and checked the statistical analysis. All authors contributed to the acquisition or interpretation of data, proof reading of the manuscript for important intellectual content, and the final approval of the version to be published. TW is the guarantor of this work and takes responsibility for the integrity of the data and the accuracy of the data analysis.

## FUNDING INFORMATION

This work was supported by the grants from the National Natural Science Foundation of China (82022011, 81970706, 82088102, 81970728), the “Shanghai Municipal Education Commission–Gaofeng Clinical Medicine Grant Support” from Shanghai Jiao Tong University School of Medicine (20171901 Round 2), the Innovative Research Team of High‐level Local Universities in Shanghai, and the Shanghai Municipal Government Grant (22Y31900300).

## CONFLICT OF INTEREST STATEMENT

None declared.

## Supporting information


Tables S1–S9

## Data Availability

All the GWAS data used in this study were public. The GWAS summary statistics for grip strength, ALM, WBLM, BF%, NAFLD, and hypertension are available from the MRC‐IEU OpenGWAS database (https://gwas.mrcieu.ac.uk/). The genetic estimates of the 53 variants associated with the composite insulin resistance phenotype were from the MR study by Qin Wang et al. (PMID: 29046328), and their genetic estimates with fasting insulin were extracted from European‐specific summary level statistics for fasting insulin available through the MAGIC website (https://www.magicinvestigators.org/). The GWAS summary statistics for type 2 diabetes are available for download at http://cg.bsc.es/70kfort2d/. The GWAS summary statistics for CHD and MI are available through the CARDIoGRAMplusC4D Consortium website (http://www.cardiogramplusc4d.org/). The GWAS summary statistics for small vessel stroke and Alzheimer's disease are available through the GWAS catalog (https://www.ebi.ac.uk/gwas/) under accession no. GCST005841 and no. GCST90027158, respectively.
