# Supplementary material for: Causal associations of sarcopenia‐related traits with cardiometabolic disease and Alzheimer's disease and the mediating role of insulin resistance: A Mendelian randomization study
Source: Aging Cell. 2023 Jul 5;22(9):e13923. doi: 10.1111/acel.13923 (PMC10497819; doi:10.1111/acel.13923)
Supplement: Supplementary file 1 — Tables S1–S9 [file ACEL-22-e13923-s001.pdf]

Causal associations of sarcopenia-related traits with cardiometabolic disease and Alzheimer's disease and the mediating role of insulin resistance: A Mendelian randomization study

Running title: Sarcopenia, insulin, and CVD outcomes

Chaojie Ye,[1,2] Lijie Kong,[1,2] Yiying Wang,[1,2] Jie Zheng,[1,2] Min Xu,[1,2] Yu Xu,[1,2] Mian Li,[1,2] Zhiyun Zhao,[1,2] Jieli Lu,[1,2] Yuhong Chen,[1,2] Weiqing Wang,[1,2] Guang Ning,[1,2] Yufang Bi,[1,2] Tiange Wang [1,2]

Author Affiliations:

[1]Department of Endocrine and Metabolic Diseases, Shanghai Institute of Endocrine and Metabolic Diseases, Ruijin Hospital, Shanghai Jiao Tong University School of Medicine, Shanghai, China;

[2]Shanghai National Clinical Research Center for Metabolic Diseases, Key Laboratory for Endocrine and Metabolic Diseases of the National Health Commission of the PR China, Shanghai Key Laboratory for Endocrine Tumor, Ruijin Hospital, Shanghai Jiao Tong University School of Medicine, Shanghai, China;

Address for Correspondence:

Tiange Wang, MD, PhD. Shanghai National Clinical Research Center for Metabolic Diseases, Shanghai Institute of Endocrine and Metabolic Diseases, Ruijin Hospital, Shanghai Jiao Tong University School of Medicine, 197 Rujin 2nd Road, Shanghai, China. E-mail: tiange.wang@shsmu.edu.cn. Phone number: 86-021-64370045.

Supplementary Material

Table of contents

**Supplementary Table 1.** UVMR estimates for the causal associations of sarcopenia-related traits with cardiometabolic diseases and Alzheimer's disease	……….….3
**Supplementary Table 2.** UVMR directional pleiotropy test and heterogeneity test for the causal associations of sarcopenia-related traits with cardiometabolic diseases and Alzheimer's disease	……….….8
**Supplementary Table 3.** UVMR estimates for the causal associations of BF% with cardiometabolic diseases and Alzheimer's disease	……….….9
**Supplementary Table 4.** MVMR estimates for the causal associations of sarcopenia-related traits with cardiometabolic diseases and Alzheimer's disease	………...10
**Supplementary Table 5.** UVMR estimates for the bi-directional causal associations of sarcopenia-related traits with insulin resistance	……….. 12
**Supplementary Table 6.** UVMR directional pleiotropy test and heterogeneity test for the bi-directional causal associations of sarcopenia-related traits with insulin resistance	………..13
**Supplementary Table 7.** UVMR estimates for the causal associations of insulin resistance with cardiometabolic diseases and Alzheimer's disease	………...14
**Supplementary Table 8.** MVMR estimates for the causal associations of insulin resistance with cardiometabolic diseases and Alzheimer's disease	………...16
**Supplementary Table 9.** MVMR estimates for the insulin resistance-adjusted causal effects of sarcopenia-related traits on cardiometabolic diseases	………...19

Supplementary Table 1. UVMR estimates for the causal associations of sarcopenia-related traits with cardiometabolic diseases and Alzheimer's disease

Exposure	Outcome	Method	No. of SNP or outlier[a]	OR (95% CI)[b]	P value	FDR q value
Grip strength	Type 2 diabetes	IVW	153	1.57 (1.13, 2.20)	0.008	0.012
		Weighted median		1.77 (1.21, 2.60)	0.003	NA
		Weighted mode		1.97 (0.77, 5.06)	0.16	NA
		MR Egger		2.45 (0.66, 9.14)	0.18	NA
		MR PRESSO	2	1.43 (1.06, 1.93)	0.022	NA
ALM		IVW	630	1.20 (1.10, 1.32)	6.42×10^{-5}	1.73×10^{-4}
		Weighted median		1.22 (1.08, 1.38)	0.001	NA
		Weighted mode		1.33 (0.99, 1.77)	0.055	NA
		MR Egger		1.17 (0.94, 1.45)	0.16	NA
		MR PRESSO	13	1.22 (1.12, 1.32)	1.94×10^{-6}	NA
WBLM		IVW	531	1.20 (1.02, 1.41)	0.032	0.047
		Weighted median		1.12 (0.90, 1.39)	0.32	NA
		Weighted mode		1.11 (0.64, 1.90)	0.72	NA
		MR Egger		1.53 (1.03, 2.28)	0.037	NA
		MR PRESSO	13	1.16 (1.01, 1.33)	0.041	NA
Walking pace		IVW	58	2.30 (1.14, 4.68)	0.021	0.032
		Weighted median		1.35 (0.62, 2.96)	0.45	NA
		Weighted mode		0.73 (0.14, 3.92)	0.72	NA
		MR Egger		0.97 (0.038, 24.47)	0.99	NA
		MR PRESSO	1	1.88 (0.99, 3.58)	0.060	NA
Grip strength	NAFLD	IVW	149	2.30 (1.02, 5.18)	0.045	0.061
		Weighted median		4.52 (1.43, 14.26)	0.010	NA
		Weighted mode		18.11 (0.80, 411.56)	0.071	NA
		MR Egger		35.62 (1.68, 754.24)	0.023	NA
		MR PRESSO	0	2.20 (0.99, 4.92)	0.056	NA
ALM		IVW	618	1.33 (1.08, 1.64)	0.006	0.011
		Weighted median		1.47 (1.05, 2.06)	0.024	NA
		Weighted mode		1.70 (0.86, 3.36)	0.13	NA

		MR Egger		1.87 (1.17, 3.00)	0.009	NA
		MR PRESSO	0	1.31 (1.07, 1.61)	0.008	NA
WBLM		IVW	523	1.28 (0.86, 1.91)	0.23	0.26
		Weighted median		1.62 (0.90, 2.93)	0.11	NA
		Weighted mode		3.64 (0.72, 18.40)	0.12	NA
		MR Egger		5.48 (2.14, 14.00)	4.14×10^{-4}	NA
		MR PRESSO	2	1.14 (0.78, 1.67)	0.50	NA
Walking pace		IVW	59	1.32 (0.21, 8.40)	0.77	0.77
		Weighted median		1.16 (0.10, 13.03)	0.91	NA
		Weighted mode		5.00 (0.011, 2120.06)	0.60	NA
		MR Egger		293.88 (0.020, 4425674.64)	0.25	NA
		MR PRESSO	1	1.68 (0.29, 9.79)	0.57	NA
Grip strength	Hypertension	IVW	149	1.32 (1.09, 1.60)	0.004	0.007
		Weighted median		1.43 (1.11, 1.83)	1.07×10^{-4}	NA
		Weighted mode		1.61 (0.84, 3.09)	0.080	NA
		MR Egger		3.06 (1.51, 6.19)	0.002	NA
		MR PRESSO	1	1.37 (1.14, 1.65)	9.68×10^{-4}	NA
ALM		IVW	618	1.12 (1.05, 1.20)	4.13×10^{-4}	0.001
		Weighted median		1.08 (1.01, 1.17)	0.040	NA
		Weighted mode		1.00 (0.84, 1.18)	0.99	NA
		MR Egger		1.12 (0.96, 1.29)	0.14	NA
		MR PRESSO	24	1.10 (1.04, 1.16)	8.18×10^{-4}	NA
WBLM		IVW	523	0.90 (0.81, 0.99)	0.037	0.052
		Weighted median		1.13 (0.99, 1.29)	0.061	NA
		Weighted mode		0.91 (0.64, 1.31)	0.63	NA
		MR Egger		1.04 (0.82, 1.32)	0.76	NA
		MR PRESSO	11	0.88 (0.80-0.96)	0.005	NA
Walking pace		IVW	59	4.43 (2.68, 7.33)	6.43×10^{-9}	3.75×10^{-8}
		Weighted median		3.93 (2.33, 6.65)	3.24×10^{-7}	NA
		Weighted mode		4.23 (1.31, 13.63)	0.019	NA
		MR Egger		0.60 (0.045, 7.93)	0.70	NA

		MR PRESSO	2	4.21 (2.66, 6.66)	8.69×10^{-8}	NA
Grip strength	CHD	IVW	149	1.42 (1.15, 1.75)	0.001	0.002
		Weighted median		1.38 (1.07, 1.78)	0.013	NA
		Weighted mode		1.77 (0.87, 3.57)	0.12	NA
		MR Egger		2.09 (0.91, 4.79)	0.085	NA
		MR PRESSO	3	1.39 (1.15, 1.69)	9.36×10^{-4}	NA
ALM		IVW	614	1.20 (1.13, 1.27)	1.39×10^{-10}	9.73×10^{-10}
		Weighted median		1.20 (1.11, 1.29)	1.63×10^{-6}	NA
		Weighted mode		1.19 (1.01, 1.39)	0.035	NA
		MR Egger		1.30 (1.14, 1.48)	8.51×10^{-5}	NA
		MR PRESSO	17	1.18 (1.12, 1.24)	2.95×10^{-10}	NA
WBLM		IVW	528	1.04 (0.94, 1.14)	0.47	0.50
		Weighted median		1.04 (0.91, 1.18)	0.57	NA
		Weighted mode		1.63 (1.00, 2.63)	0.049	NA
		MR Egger		1.01 (0.80, 1.28)	0.94	NA
		MR PRESSO	10	1.11 (1.02, 1.22)	0.020	NA
Walking pace		IVW	59	2.73 (1.84, 4.05)	6.96×10^{-7}	3.05×10^{-6}
		Weighted median		2.50 (1.56, 4.02)	1.53×10^{-4}	NA
		Weighted mode		2.49 (0.79, 7.86)	0.13	NA
		MR Egger		1.76 (0.25, 12.54)	0.57	NA
		MR PRESSO	1	2.50 (1.74, 3.60)	6.80×10^{-6}	NA
Grip strength	MI	IVW	148	1.45 (1.15, 1.82)	0.002	0.003
		Weighted median		1.28 (0.98, 1.68)	0.067	NA
		Weighted mode		0.86 (0.38, 1.97)	0.73	NA
		MR Egger		2.67 (1.08, 6.60)	0.035	NA
		MR PRESSO	2	1.47 (1.18, 1.82)	6.44×10^{-4}	NA
ALM		IVW	611	1.18 (1.11, 1.25)	4.08×10^{-8}	2.04×10^{-7}
		Weighted median		1.24 (1.14, 1.35)	2.70×10^{-7}	NA
		Weighted mode		1.40 (1.18, 1.65)	9.72×10^{-5}	NA
		MR Egger		1.28 (1.11, 1.46)	4.88×10^{-4}	NA
		MR PRESSO	7	1.17 (1.11, 1.24)	1.58×10^{-8}	NA

WBLM		IVW	526	1.03 (0.93, 1.14)	0.53	0.54
		Weighted median		1.05 (0.91, 1.20)	0.52	NA
		Weighted mode		1.59 (0.93, 2.72)	0.089	NA
		MR Egger		1.05 (0.82, 1.34)	0.72	NA
		MR PRESSO	6	1.09 (0.99, 1.20)	0.073	NA
Walking pace		IVW	59	2.47 (1.63, 3.73)	1.82×10^{-5}	5.78×10^{-5}
		Weighted median		2.64 (1.59, 4.38)	1.85×10^{-4}	NA
		Weighted mode		6.22 (1.47, 26.31)	0.016	NA
		MR Egger		1.41 (0.18, 10.75)	0.74	NA
		MR PRESSO	1	2.34 (1.59, 3.44)	5.94×10^{-5}	NA
Grip strength	Small vessel stroke	IVW	131	1.25 (0.86, 1.81)	0.23	0.26
		Weighted median		1.32 (0.83, 2.10)	0.24	NA
		Weighted mode		1.14 (0.35, 3.74)	0.83	NA
		MR Egger		3.25 (0.55, 19.22)	0.20	NA
		MR PRESSO	2	1.34 (0.95, 1.89)	0.10	NA
ALM		IVW	536	1.25 (1.15, 1.37)	8.15×10^{-7}	3.17×10^{-6}
		Weighted median		1.33 (1.17, 1.52)	2.22×10^{-5}	NA
		Weighted mode		1.41 (1.04, 1.89)	0.026	NA
		MR Egger		1.55 (1.23, 1.94)	2.11×10^{-4}	NA
		MR PRESSO	3	1.25 (1.15, 1.36)	6.64×10^{-7}	NA
WBLM		IVW	471	1.29 (1.10, 1.52)	0.002	0.004
		Weighted median		1.31 (1.03, 1.65)	0.027	NA
		Weighted mode		1.47 (0.84, 2.60)	0.18	NA
		MR Egger		1.88 (1.23, 2.89)	0.004	NA
		MR PRESSO	6	1.27 (1.10, 1.48)	0.002	NA
Walking pace		IVW	52	1.96 (0.91, 4.23)	0.087	0.11
		Weighted median		1.41 (0.55, 3.65)	0.47	NA
		Weighted mode		1.00 (0.11, 9.48)	0.99	NA
		MR Egger		2.65 (0.016, 427.18)	0.71	NA
		MR PRESSO	0	1.98 (0.93, 4.23)	0.083	NA
Grip strength	Alzheimer's	IVW	146	1.10 (0.91, 1.33)	0.34	0.37

	disease	Weighted median		1.15 (0.93, 1.42)	0.19	NA
		Weighted mode		1.19 (0.75, 1.90)	0.46	NA
		MR Egger		1.23 (0.60, 2.52)	0.58	NA
		MR PRESSO	3	1.12 (0.95, 1.33)	0.18	NA
ALM		IVW	591	1.10 (1.05, 1.15)	5.10×10^{-5}	1.49×10^{-4}
		Weighted median		1.08 (1.02, 1.15)	0.008	NA
		Weighted mode		1.06 (0.93, 1.21)	0.39	NA
		MR Egger		1.16 (1.04, 1.28)	0.005	NA
		MR PRESSO	10	1.09 (1.05, 1.13)	3.47×10^{-5}	NA
WBLM		IVW	521	1.28 (1.19, 1.38)	9.34×10^{-11}	8.17×10^{-10}
		Weighted median		1.24 (1.12, 1.37)	3.93×10^{-5}	NA
		Weighted mode		1.23 (1.00, 1.52)	0.051	NA
		MR Egger		1.28 (1.07, 1.53)	0.008	NA
		MR PRESSO	8	1.28 (1.20, 1.38)	3.82×10^{-12}	NA
Walking pace		IVW	55	0.73 (0.48, 1.10)	0.13	0.16
		Weighted median		0.62 (0.39, 0.97)	0.036	NA
		Weighted mode		0.46 (0.14, 1.52)	0.21	NA
		MR Egger		4.07 (0.67, 24.73)	0.13	NA
		MR PRESSO	3	0.65 (0.46, 0.93)	0.023	NA

[a] The numbers of SNPs used as instrumental variables in the IVW, the weighted median, the weighted mode, and the MR Egger methods or the number of outliers identified and excluded in the MR PRESSO method.

[b] ORs (95% CIs) represent risks for cardiometabolic diseases and Alzheimer's disease associated with each 1-SD lower grip strength, ALM, WBLM, and a shift of one category to a lower walking pace (brisk, steady/average, slow).

Abbreviations: ALM, appendicular lean mass; CHD, coronary heart disease; CI, confidence interval; FDR, false discovery rate; IVW, inverse-variance weighted; MI, myocardial infarction; MR, Mendelian randomization; NA, not applicable; NAFLD, non-alcoholic fatty liver disease; OR, odds ratio; PRESSO, pleiotropy residual sum and outlier; SD, standard deviation; SNP, single nucleotide polymorphism; UVMR, univariable Mendelian randomization; WBLM, whole-body lean mass.

Supplementary Table 2. UVMR directional pleiotropy test and heterogeneity test for the causal associations of sarcopenia-related traits with cardiometabolic diseases and Alzheimer's disease

Exposure	Outcome	Directional pleiotropy test			Heterogeneity test		
		Egger intercept	SE	P value	Q statistic	Q df	Q p value
Grip strength	Type 2 diabetes	0.005	0.008	0.50	262.57	152	6.51×10^{-8}
ALM		-0.001	0.002	0.76	1086.15	629	6.75×10^{-27}
WBLM		0.004	0.003	0.19	970.94	530	2.45×10^{-28}
Walking pace		-0.008	0.015	0.59	109.75	57	3.40×10^{-5}
Grip strength	NAFLD	0.033	0.018	0.070	162.51	148	0.20
ALM		0.008	0.005	0.12	623.97	617	0.41
WBLM		0.022	0.006	0.001	643.89	522	0.002
Walking pace		0.050	0.044	0.27	79.85	58	0.030
Grip strength	Hypertension	0.010	0.004	0.017	209.82	148	6.29×10^{-4}
ALM		-0.000	0.002	0.93	1450.29	617	6.07×10^{-69}
WBLM		0.001	0.002	0.52	992.22	521	9.74×10^{-32}
Walking pace		-0.018	0.012	0.13	139.49	58	1.15×10^{-8}
Grip strength	CHD	0.005	0.005	0.35	275.90	148	9.09×10^{-10}
ALM		0.002	0.001	0.18	1113.83	613	1.52×10^{-31}
WBLM		-0.000	0.002	0.81	949.22	527	1.37×10^{-26}
Walking pace		-0.004	0.009	0.66	95.97	58	0.001
Grip strength	MI	0.007	0.005	0.17	267.73	147	4.56×10^{-9}
ALM		0.002	0.002	0.20	1009.95	610	2.98×10^{-22}
WBLM		0.000	0.002	0.92	841.13	525	4.92×10^{-17}
Walking pace		-0.005	0.009	0.58	85.11	58	0.012
Grip strength	Small vessel stroke	0.011	0.010	0.29	197.86	130	1.16×10^{-4}
ALM		0.005	0.002	0.052	677.71	535	2.61×10^{-5}
WBLM		0.005	0.003	0.063	623.94	470	2.33×10^{-6}
Walking pace		0.003	0.023	0.91	82.84	51	0.003
Grip strength	Alzheimer's disease	0.001	0.004	0.75	323.10	145	1.34×10^{-15}
ALM		0.001	0.001	0.25	958.88	590	4.81×10^{-20}
WBLM		-0.000	0.001	0.96	789.84	520	1.88×10^{-13}
Walking pace		0.016	0.008	0.061	125.92	54	1.12×10^{-7}

Abbreviations: ALM, appendicular lean mass; CHD, coronary heart disease; MI, myocardial infarction; NAFLD, non-alcoholic fatty liver disease; SE, standard error; UVMR, univariable Mendelian randomization; WBLM, whole-body lean mass.

Supplementary Table 3. UVMR estimates for the causal associations of BF% with cardiometabolic diseases and Alzheimer's disease

Exposure	Outcome	Method	No. of SNP	OR (95% CI)[a]	P value	FDR q value
BF%	Type 2 diabetes	IVW	375	1.44 (1.17, 1.76)	4.54×10^{-4}	0.001
		Weighted median		1.59 (1.27, 2.00)	6.56×10^{-5}	NA
		Weighted mode		1.91 (1.07, 3.41)	0.029	NA
		MR Egger		1.55 (0.81, 2.96)	0.19	NA
BF%	NAFLD	IVW	372	1.96 (1.21, 3.15)	0.006	0.011
		Weighted median		1.95 (1.00, 3.79)	0.051	NA
		Weighted mode		2.80 (0.54, 14.50)	0.22	NA
		MR Egger		6.01 (1.28, 28.26)	0.024	NA
BF%	Hypertension	IVW	372	1.83 (1.61, 2.09)	2.70×10^{-20}	9.45×10^{-19}
		Weighted median		2.01 (1.74, 2.32)	7.31×10^{-21}	NA
		Weighted mode		2.15 (1.33, 3.49)	0.002	NA
		MR Egger		1.72 (1.13, 2.62)	0.011	NA
BF%	CHD	IVW	374	1.58 (1.41, 1.77)	7.40×10^{-15}	8.63×10^{-14}
		Weighted median		1.70 (1.48, 1.95)	2.30×10^{-14}	NA
		Weighted mode		1.88 (1.37, 2.58)	9.56×10^{-5}	NA
		MR Egger		1.67 (1.15, 2.43)	0.007	NA
BF%	MI	IVW	372	1.62 (1.44, 1.82)	1.23×10^{-15}	2.15×10^{-14}
		Weighted median		1.81 (1.55, 2.11)	7.70×10^{-14}	NA
		Weighted mode		1.95 (1.38, 2.75)	1.80×10^{-4}	NA
		MR Egger		1.71 (1.17, 2.52)	0.006	NA
BF%	Small vessel stroke	IVW	349	1.13 (0.95, 1.36)	0.16	0.19
		Weighted median		1.04 (0.82, 1.33)	0.73	NA
		Weighted mode		0.66 (0.30, 1.46)	0.30	NA
		MR Egger		1.29 (0.71, 2.36)	0.41	NA
BF%	Alzheimer's disease	IVW	374	0.81 (0.74, 0.89)	9.19×10^{-6}	3.22×10^{-5}
		Weighted median		0.82 (0.72, 0.92)	7.94×10^{-4}	NA
		Weighted mode		0.80 (0.59, 1.07)	0.14	NA
		MR Egger		0.77 (0.57, 1.04)	0.087	NA

[a] ORs (95% CIs) represent risks for cardiometabolic diseases and Alzheimer's disease associated with each 1-SD lower BF%.

Abbreviations: BF%, body fat percentage; CHD, coronary heart disease; CI, confidence interval; FDR, false discovery rate; IVW, inverse-variance weighted; MI, myocardial infarction; MR, Mendelian randomization; NA, not available; NAFLD, non-alcoholic fatty liver disease; OR, odds ratio; SD, standard deviation; SNP, single nucleotide polymorphism; UVMR, univariable Mendelian randomization.

Supplementary Table 4. MVMR estimates for the causal associations of sarcopenia-related traits with cardiometabolic diseases and Alzheimer's disease

Exposure	Outcome	Adjusted for	Method	OR (95% CI)[a]	P value
Grip strength	Type 2 diabetes	BF%, Physical activity (device-measured)	MV-IVW	1.21 (0.77, 1.89)	0.41
			MVMR Egger	1.40 (0.73, 2.68)	0.31
ALM			MV-IVW	1.17 (1.06, 1.30)	0.003
			MVMR Egger	1.24 (1.02, 1.52)	0.035
WBLM			MV-IVW	1.44 (1.17, 1.76)	4.50×10^{-4}
			MVMR Egger	1.98 (1.39, 2.83)	1.77×10^{-4}
Walking pace			MV-IVW	3.30 (1.15, 9.48)	0.027
			MVMR Egger	3.25 (0.95, 11.05)	0.059
Grip strength	NAFLD	BF%, Physical activity (device-measured)	MV-IVW	3.23 (1.09, 9.58)	0.034
			MVMR Egger	14.57 (3.06, 69.40)	7.70×10^{-4}
ALM			MV-IVW	1.34 (1.06, 1.71)	0.016
			MVMR Egger	1.98 (1.27, 3.10)	0.003
WBLM			MV-IVW	1.70 (1.09, 2.65)	0.020
			MVMR Egger	3.74 (1.73, 8.06)	7.66×10^{-4}
Walking pace			MV-IVW	1.94 (0.16, 24.24)	0.61
			MVMR Egger	2.06 (0.10, 41.01)	0.64
Grip strength	Hypertension	BF%, Physical activity (device-measured)	MV-IVW	1.12 (0.84, 1.49)	0.45
			MVMR Egger	1.42 (0.93, 2.15)	0.10
ALM			MV-IVW	1.02 (0.95, 1.10)	0.55
			MVMR Egger	1.05 (0.91, 1.20)	0.52
WBLM			MV-IVW	0.97 (0.86, 1.10)	0.63
			MVMR Egger	1.13 (0.91, 1.40)	0.26
Walking pace			MV-IVW	3.11 (1.58, 6.12)	0.001
			MVMR Egger	3.35 (1.50, 7.47)	0.003
Grip strength	CHD	BF%, Physical activity (device-measured)	MV-IVW	1.12 (0.86, 1.45)	0.41
			MVMR Egger	1.48 (1.02, 2.16)	0.040
ALM			MV-IVW	1.11 (1.04, 1.18)	0.002
			MVMR Egger	1.26 (1.12, 1.42)	1.38×10^{-4}
WBLM			MV-IVW	1.14 (1.01, 1.27)	0.027
			MVMR Egger	1.30 (1.07, 1.59)	0.009
Walking pace			MV-IVW	1.50 (0.82, 2.73)	0.19
			MVMR Egger	1.83 (0.91, 3.68)	0.090
Grip strength	MI	BF%, Physical activity (device-measured)	MV-IVW	1.16 (0.88, 1.52)	0.29
			MVMR Egger	1.41 (0.96, 2.09)	0.082
ALM			MV-IVW	1.08 (1.01, 1.15)	0.023
			MVMR Egger	1.25 (1.10, 1.42)	5.84×10^{-4}
WBLM			MV-IVW	1.16 (1.03, 1.31)	0.016
			MVMR Egger	1.31 (1.06, 1.62)	0.013
Walking pace			MV-IVW	1.51 (0.81, 2.80)	0.19
			MVMR Egger	1.63 (0.79, 3.35)	0.18
Grip strength	Small vessel stroke	BF%, Physical activity (device-measured)	MV-IVW	1.23 (0.82, 1.85)	0.32
			MVMR Egger	1.67 (0.91, 3.06)	0.096
ALM			MV-IVW	1.22 (1.10, 1.35)	1.93×10^{-4}
			MVMR Egger	1.52 (1.23, 1.86)	7.29×10^{-5}
WBLM			MV-IVW	1.42 (1.17, 1.73)	4.96×10^{-4}
			MVMR Egger	1.86 (1.29, 2.68)	9.40×10^{-4}
Walking pace			MV-IVW	1.44 (0.56, 3.72)	0.45
			MVMR Egger	1.72 (0.56, 5.23)	0.34
Grip strength	Alzheimer's disease	BF%, Physical activity (device-measured)	MV-IVW	1.11 (0.90, 1.35)	0.32
			MVMR Egger	1.10 (0.82, 1.47)	0.53
ALM			MV-IVW	1.12 (1.07, 1.18)	6.95×10^{-6}
			MVMR Egger	1.14 (1.04, 1.25)	0.005
WBLM			MV-IVW	1.22 (1.11, 1.33)	1.50×10^{-5}

			MVMR Egger	1.28 (1.09, 1.49)	0.002
Walking pace			MV-IVW	1.66 (1.02, 2.70)	0.042
			MVMR Egger	1.48 (0.84, 2.61)	0.18

[a] ORs (95% CIs) represent risks for cardiometabolic diseases and Alzheimer's disease associated with each 1-SD lower grip strength, ALM, WBLM, and a shift of one category to a lower walking pace (brisk, steady/average, slow).
Abbreviations: ALM, appendicular lean mass; BF%, body fat percentage; CHD, coronary heart disease; CI, confidence interval; MI, myocardial infarction; MV-IVW, multivariable inverse-variance weighted; MVMR, multivariable Mendelian randomization; MVMR Egger, multivariable Mendelian randomization Egger; NAFLD, non-alcoholic fatty liver disease; OR, odds ratio; SD, standard deviation; WBLM, whole-body lean mass.

Supplementary Table 5. UVMR estimates for the bi-directional causal associations of sarcopenia-related traits with insulin resistance

Exposure	Outcome	Method	No. of SNP or outlier[a]	β (95% CI)[b]	P value
Grip strength	Insulin resistance (fasting insulin)	IVW	154	0.064 (0.016, 0.113)	0.009
		Weighted median		0.045 (-0.008, 0.097)	0.094
		Weighted mode		0.051 (-0.088, 0.191)	0.47
		MR Egger		0.197 (0.009, 0.386)	0.042
		MR PRESSO	6	0.067 (0.027, 0.108)	0.001
ALM		IVW	618	0.027 (0.011, 0.042)	7.45×10^{-4}
		Weighted median		0.049 (0.031, 0.067)	4.76×10^{-8}
		Weighted mode		0.071 (0.034, 0.108)	1.76×10^{-4}
		MR Egger		0.063 (0.027, 0.099)	6.05×10^{-4}
		MR PRESSO	28	0.032 (0.020, 0.044)	4.82×10^{-7}
WBLM		IVW	534	0.081 (0.056, 0.106)	1.47×10^{-10}
		Weighted median		0.090 (0.059, 0.121)	1.09×10^{-8}
		Weighted mode		0.108 (0.052, 0.163)	1.52×10^{-4}
		MR Egger		0.125 (0.065, 0.184)	4.97×10^{-5}
		MR PRESSO	24	0.080 (0.060, 0.100)	7.66×10^{-14}
Walking pace		IVW	59	0.026 (-0.074, 0.127)	0.61
		Weighted median		-0.021 (-0.137, 0.095)	0.72
		Weighted mode		-0.109 (-0.443, 0.225)	0.53
		MR Egger		-0.041 (-0.533, 0.452)	0.87
		MR PRESSO	2	0.024 (-0.069, 0.116)	0.62
Insulin resistance (fasting insulin)	Grip strength	IVW	32	0.080 (-0.001, 0.162)	0.053
		Weighted median		0.097 (0.033, 0.160)	0.003
		Weighted mode		0.100 (0.015, 0.184)	0.028
		MR Egger		0.139 (-0.125, 0.403)	0.31
	ALM	IVW	33	0.348 (-0.016, 0.712)	0.061
		Weighted median		0.247 (0.083, 0.411)	0.003
		Weighted mode		0.212 (-0.157, 0.580)	0.27
		MR Egger		0.699 (-0.409, 1.806)	0.23
	WBLM	IVW	32	0.048 (-0.132, 0.229)	0.60
		Weighted median		0.109 (0.040, 0.177)	0.002
		Weighted mode		0.158 (0.077, 0.239)	6.12×10^{-4}
		MR Egger		0.401 (-0.173, 0.975)	0.18
	Walking pace	IVW	38	0.056 (-0.006, 0.118)	0.075
		Weighted median		0.033 (-0.018, 0.083)	0.20
		Weighted mode		0.031 (-0.040, 0.103)	0.39
		MR Egger		-0.016 (-0.203, 0.170)	0.86

[a] The numbers of SNPs used as instrumental variables in the IVW, the weighted median, the weighted mode, and the MR Egger methods or the number of outliers identified and excluded in the MR PRESSO method.

[b] β estimates (95% CIs) represent the associations of each 1-SD lower grip strength, ALM, WBLM, and a shift of one category to a lower walking pace (brisk, steady/average, slow) with insulin resistance (log-transformed pmol/L fasting insulin), or vice versa.

Abbreviations: ALM, appendicular lean mass; CI, confidence interval; IVW, inverse-variance weighted; MR, Mendelian randomization; PRESSO, pleiotropy residual sum and outlier; SD, standard deviation; SNP, single nucleotide polymorphism; UVMR, univariable Mendelian randomization; WBLM, whole-body lean mass.

Supplementary Table 6. UVMR directional pleiotropy test and heterogeneity test for the bi-directional causal associations of sarcopenia-related traits with insulin resistance

		Directional pleiotropy test			Heterogeneity test		
Exposure	Outcome	Egger intercept	SE	P value	Q statistic	Q df	Q p value
Grip strength	Insulin resistance (fasting insulin)	0.002	0.001	0.15	339.26	153	3.77×10^{-16}
ALM		0.001	0.000	0.028	1884.76	617	2.32×10^{-128}
WBLM		0.001	0.000	0.12	1391.72	533	6.24×10^{-78}
Walking pace		-0.001	0.002	0.79	132.68	58	8.81×10^{-8}
Insulin resistance (fasting insulin)	Grip strength	-0.001	0.002	0.65	157.79	31	6.39×10^{-19}
	ALM	-0.006	0.010	0.52	2122.49	32	$0.00\times10^{+00}$
	WBLM	-0.006	0.005	0.21	1117.75	31	4.04×10^{-215}
	Walking pace	0.001	0.002	0.42	177.97	37	2.43×10^{-20}

Abbreviations: ALM, appendicular lean mass; SE, standard error; UVMR, univariable Mendelian randomization; WBLM, whole-body lean mass.

Supplementary Table 7. UVMR estimates for the causal associations of insulin resistance with cardiometabolic diseases and Alzheimer's disease

Insulin resistance trait	Outcome	Method	No. of SNP	OR (95% CI)[a]	P value
Insulin resistance (fasting insulin)	Type 2 diabetes	IVW	47	23.98 (10.88, 52.86)	3.32×10^{-15}
		Weighted median		37.01 (16.53, 82.90)	1.66×10^{-18}
		Weighted mode		45.53 (7.40, 279.99)	1.56×10^{-4}
		MR Egger		21.78 (4.01, 118.38)	8.69×10^{-4}
		MR PRESSO	2	38.87 (21.65, 69.78)	8.67×10^{-16}
Insulin resistance (fasting insulin)	NAFLD	IVW	47	17.52 (3.30, 92.93)	7.70×10^{-4}
		Weighted median		8.73 (1.06, 72.05)	0.044
		Weighted mode		26.09 (0.01, 245649.80)	0.49
		MR Egger		10.49 (0.30, 368.03)	0.20
		MR PRESSO	0	17.52 (3.30, 92.93)	0.002
Insulin resistance (fasting insulin)	Hypertension	IVW	47	3.12 (2.16, 4.49)	1.09×10^{-9}
		Weighted median		3.86 (2.42, 6.16)	1.46×10^{-8}
		Weighted mode		3.88 (1.74, 8.66)	0.002
		MR Egger		2.30 (1.06, 4.99)	0.041
		MR PRESSO	1	3.26 (2.30, 4.63)	3.85×10^{-8}
Insulin resistance (fasting insulin)	CHD	IVW	47	2.93 (2.00, 4.30)	3.59×10^{-8}
		Weighted median		3.52 (2.21, 5.60)	1.03×10^{-7}
		Weighted mode		3.70 (1.11, 12.30)	0.038
		MR Egger		1.65 (0.74, 3.67)	0.23
		MR PRESSO	0	2.93 (2.00, 4.30)	1.56×10^{-6}
Insulin resistance (fasting insulin)	MI	IVW	47	2.54 (1.70, 3.79)	5.62×10^{-6}
		Weighted median		1.88 (1.13, 3.12)	0.014
		Weighted mode		2.69 (0.81, 8.96)	0.11
		MR Egger		1.25 (0.54, 2.87)	0.60
		MR PRESSO	1	2.43 (1.66, 3.55)	4.04×10^{-5}
Insulin resistance (fasting insulin)	Small vessel stroke	IVW	44	2.35 (1.24, 4.45)	0.009
		Weighted median		1.44 (0.62, 3.36)	0.40
		Weighted mode		1.79 (0.63, 5.11)	0.28
		MR Egger		0.92 (0.24, 3.55)	0.91
		MR PRESSO	1	2.90 (1.58, 5.33)	0.001
Insulin resistance (fasting insulin)	Alzheimer's disease	IVW	47	1.12 (0.86, 1.44)	0.40
		Weighted median		1.38 (0.97, 1.98)	0.073
		Weighted mode		1.39 (0.37, 5.20)	0.63
		MR Egger		1.22 (0.70, 2.12)	0.48
		MR PRESSO	0	1.12 (0.86, 1.44)	0.40
Insulin resistance phenotype	Type 2 diabetes	IVW	47	4.24 (2.92, 6.16)	2.68×10^{-14}
		Weighted median		3.20 (2.13, 4.82)	2.51×10^{-8}
		Weighted mode		2.10 (1.35, 3.29)	0.002
		MR Egger		2.20 (1.09, 4.44)	0.032
		MR PRESSO	3	5.60 (3.79, 8.30)	6.62×10^{-11}
Insulin resistance phenotype	NAFLD	IVW	47	3.61 (1.57, 8.32)	0.003
		Weighted median		2.47 (0.85, 7.18)	0.096
		Weighted mode		1.31 (0.39, 4.37)	0.66
		MR Egger		1.81 (0.34, 9.65)	0.49
		MR PRESSO	1	4.05 (1.87, 8.76)	9.15×10^{-4}
Insulin resistance phenotype	Hypertension	IVW	47	1.60 (1.32, 1.95)	2.21×10^{-6}
		Weighted median		1.58 (1.23, 2.02)	3.23×10^{-4}
		Weighted mode		1.17 (0.90, 1.54)	0.25
		MR Egger		1.10 (0.76, 1.61)	0.61

		MR PRESSO	1	1.82 (1.47, 2.25)	1.93×10^{-6}
Insulin resistance phenotype	CHD	IVW	47	1.82 (1.55, 2.14)	2.78×10^{-13}
		Weighted median		1.71 (1.36, 2.14)	2.90×10^{-6}
		Weighted mode		1.77 (1.34, 2.35)	2.20×10^{-4}
		MR Egger		1.70 (1.23, 2.35)	0.003
		MR PRESSO	0	1.82 (1.55, 2.14)	3.19×10^{-9}
Insulin resistance phenotype	MI	IVW	47	1.79 (1.51, 2.11)	6.29×10^{-12}
		Weighted median		1.73 (1.33, 2.25)	4.20×10^{-5}
		Weighted mode		1.69 (1.29, 2.20)	3.70×10^{-4}
		MR Egger		1.83 (1.31, 2.55)	9.67×10^{-4}
		MR PRESSO	0	1.79 (1.51, 2.11)	1.41×10^{-8}
Insulin resistance phenotype	Small vessel stroke	IVW	44	1.60 (1.20, 2.15)	0.002
		Weighted median		1.41 (0.93, 2.14)	0.10
		Weighted mode		1.38 (0.90, 2.13)	0.14
		MR Egger		1.40 (0.79, 2.50)	0.25
		MR PRESSO	0	1.60 (1.20, 2.15)	0.003
Insulin resistance phenotype	Alzheimer’s disease	IVW	47	1.00 (0.89, 1.13)	0.98
		Weighted median		0.94 (0.78, 1.14)	0.55
		Weighted mode		0.94 (0.78, 1.13)	0.50
		MR Egger		0.88 (0.69, 1.11)	0.28
		MR PRESSO	0	1.00 (0.89, 1.13)	0.98

[a] ORs (95% CIs) represent risks for cardiometabolic diseases and Alzheimer’s disease associated with each 1-log unit higher insulin resistance (pmol/L fasting insulin) or each 1-SD higher insulin resistance phenotype equivalent to 55% higher geometric mean of fasting insulin, 0.89 mmol/L higher triglycerides, and 0.46 mmol/L lower HDL-C.

Abbreviations: CHD, coronary heart disease; CI, confidence interval; HDL-C, high-density lipoprotein cholesterol; IVW, inverse-variance weighted; MI, myocardial infarction; MR, Mendelian randomization; NAFLD, non-alcoholic fatty liver disease; OR, odds ratio; SD, standard deviation; SNP, single nucleotide polymorphism; UVMR, univariable Mendelian randomization.

Supplementary Table 8. MVMR estimates for the causal associations of insulin resistance with cardiometabolic diseases and Alzheimer's disease

Insulin resistance trait	Outcome	Sarcopenia-related trait adjusted for	Method	β (SE)[a]	OR (95% CI)[a]	P value	F-statistic
Insulin resistance (fasting insulin)	Type 2 diabetes	Grip strength	MV-IVW	2.399 (0.350)	11.01 (5.55, 21.84)	6.80×10^{-12}	26
			MVMR Egger	2.622 (0.463)	13.77 (5.55, 34.13)	1.50×10^{-8}	
		ALM	MV-IVW	1.940 (0.211)	6.96 (4.60, 10.52)	3.55×10^{-20}	50
			MVMR Egger	1.956 (0.211)	7.07 (4.67, 10.69)	2.05×10^{-20}	
		WBLM	MV-IVW	2.253 (0.254)	9.52 (5.79, 15.65)	6.53×10^{-19}	42
			MVMR Egger	2.601 (0.350)	13.48 (6.80, 26.75)	9.90×10^{-14}	
		Walking pace	MV-IVW	2.931 (0.314)	18.74 (10.13, 34.67)	9.46×10^{-21}	45
			MVMR Egger	2.900 (0.313)	18.17 (9.84, 33.56)	1.91×10^{-20}	
Insulin resistance (fasting insulin)	NAFLD	Grip strength	MV-IVW	2.685 (0.753)	14.66 (3.35, 64.19)	3.65×10^{-4}	18
			MVMR Egger	4.028 (0.983)	56.18 (8.17, 386.11)	4.20×10^{-5}	
		ALM	MV-IVW	2.316 (0.502)	10.13 (3.79, 27.10)	3.96×10^{-6}	14
			MVMR Egger	2.264 (0.503)	9.62 (3.59, 25.82)	6.89×10^{-6}	
		WBLM	MV-IVW	1.085 (0.639)	2.96 (0.85, 10.36)	0.089	13
			MVMR Egger	1.642 (0.878)	5.17 (0.92, 28.88)	0.061	
		Walking pace	MV-IVW	2.635 (0.781)	13.95 (3.02, 64.43)	7.39×10^{-4}	13
			MVMR Egger	2.595 (0.788)	13.40 (2.86, 62.83)	9.93×10^{-4}	
Insulin resistance (fasting insulin)	Hypertension	Grip strength	MV-IVW	1.181 (0.173)	3.26 (2.32, 4.57)	8.32×10^{-12}	27
			MVMR Egger	1.241 (0.228)	3.46 (2.21, 5.41)	5.45×10^{-8}	
		ALM	MV-IVW	0.472 (0.157)	1.60 (1.18, 2.18)	0.003	19
			MVMR Egger	0.480 (0.157)	1.62 (1.19, 2.20)	0.002	
		WBLM	MV-IVW	0.360 (0.163)	1.43 (1.04, 1.97)	0.027	16
			MVMR Egger	0.440 (0.224)	1.55 (1.00, 2.41)	0.049	
		Walking pace	MV-IVW	1.038 (0.194)	2.82 (1.93, 4.13)	9.15×10^{-8}	35
			MVMR Egger	1.026 (0.196)	2.79 (1.90, 4.10)	1.65×10^{-7}	
Insulin resistance (fasting insulin)	CHD	Grip strength	MV-IVW	0.986 (0.203)	2.68 (1.80, 3.99)	1.16×10^{-6}	16
			MVMR Egger	1.263 (0.268)	3.54 (2.09, 5.99)	2.52×10^{-6}	
		ALM	MV-IVW	0.476 (0.133)	1.61 (1.24, 2.09)	3.46×10^{-4}	23

			MVMR Egger	0.462 (0.133)	1.59 (1.22, 2.06)	5.17×10^{-4}	
		WBLM	MV-IVW	0.533 (0.155)	1.70 (1.26, 2.31)	5.72×10^{-4}	16
			MVMR Egger	0.675 (0.216)	1.96 (1.29, 3.00)	0.002	
		Walking pace	MV-IVW	1.023 (0.172)	2.78 (1.98, 3.90)	2.89×10^{-9}	29
			MVMR Egger	1.026 (0.174)	2.79 (1.99, 3.92)	3.46×10^{-9}	
Insulin resistance (fasting insulin)	MI	Grip strength	MV-IVW	0.920 (0.223)	2.51 (1.62, 3.89)	3.81×10^{-5}	12
			MVMR Egger	1.083 (0.297)	2.95 (1.65, 5.29)	2.64×10^{-4}	
		ALM	MV-IVW	0.446 (0.140)	1.56 (1.19, 2.06)	0.001	17
			MVMR Egger	0.431 (0.140)	1.54 (1.17, 2.02)	0.002	
		WBLM	MV-IVW	0.508 (0.164)	1.66 (1.20, 2.29)	0.002	15
			MVMR Egger	0.654 (0.228)	1.92 (1.23, 3.01)	0.004	
		Walking pace	MV-IVW	0.824 (0.190)	2.28 (1.57, 3.31)	1.37×10^{-5}	17
			MVMR Egger	0.832 (0.191)	2.30 (1.58, 3.34)	1.28×10^{-5}	
Insulin resistance (fasting insulin)	Small vessel stroke	Grip strength	MV-IVW	0.806 (0.340)	2.24 (1.15, 4.36)	0.018	17
			MVMR Egger	0.977 (0.457)	2.66 (1.09, 6.50)	0.032	
		ALM	MV-IVW	0.301 (0.216)	1.35 (0.88, 2.06)	0.17	11
			MVMR Egger	0.288 (0.216)	1.33 (0.87, 2.04)	0.18	
		WBLM	MV-IVW	0.383 (0.251)	1.47 (0.90, 2.40)	0.13	15
			MVMR Egger	0.451 (0.352)	1.57 (0.79, 3.13)	0.20	
		Walking pace	MV-IVW	0.776 (0.315)	2.17 (1.17, 4.03)	0.014	14
			MVMR Egger	0.770 (0.317)	2.16 (1.16, 4.02)	0.015	
Insulin resistance (fasting insulin)	Alzheimer's disease	Grip strength	MV-IVW	-0.062 (0.183)	0.94 (0.66, 1.35)	0.74	17
			MVMR Egger	0.134 (0.241)	1.14 (0.71, 1.83)	0.58	
		ALM	MV-IVW	0.179 (0.114)	1.20 (0.96, 1.50)	0.12	18
			MVMR Egger	0.166 (0.115)	1.18 (0.94, 1.48)	0.15	
		WBLM	MV-IVW	0.029 (0.119)	1.03 (0.82, 1.30)	0.81	19
			MVMR Egger	0.219 (0.165)	1.25 (0.90, 1.72)	0.18	
		Walking pace	MV-IVW	0.214 (0.167)	1.24 (0.89, 1.72)	0.20	13
			MVMR Egger	0.221 (0.168)	1.25 (0.90, 1.73)	0.19	

[a] β estimates (SEs) and ORs (95% CIs) represent risks for cardiometabolic diseases and Alzheimer's disease associated with 1-log unit higher insulin resistance (pmol/L fasting insulin).

Abbreviations: ALM, appendicular lean mass; CHD, coronary heart disease; CI, confidence interval; MI, myocardial infarction; MV-IVW, multivariable inverse-variance weighted; MVMR, multivariable Mendelian randomization; MVMR Egger, multivariable Mendelian randomization Egger; NAFLD, non-alcoholic fatty liver disease; OR, odds ratio; SD, standard deviation; SE, standard error; WBLM, whole-body lean mass.

Supplementary Table 9. MVMR estimates for the insulin resistance-adjusted causal effects of sarcopenia-related traits on cardiometabolic diseases

Outcome	Exposure	Adjusted for	Method	OR (95% CI)[a]	P value
Type 2 diabetes	Grip strength	Insulin resistance (fasting insulin)	MV-IVW	1.35 (0.94, 1.95)	0.11
			MVMR Egger	1.38 (0.95, 1.99)	0.091
	ALM		MV-IVW	1.13 (1.03, 1.24)	0.007
			MVMR Egger	1.01 (0.81, 1.25)	0.94
	WBLM		MV-IVW	0.99 (0.84, 1.17)	0.92
			MVMR Egger	1.00 (0.85, 1.19)	0.99
NAFLD	Grip strength	Insulin resistance (fasting insulin)	MV-IVW	1.94 (0.87, 4.34)	0.11
			MVMR Egger	2.15 (0.97, 4.80)	0.061
	ALM		MV-IVW	1.25 (1.01, 1.55)	0.041
			MVMR Egger	1.68 (1.04, 2.73)	0.035
Hypertension	Grip strength	Insulin resistance (fasting insulin)	MV-IVW	1.23 (1.02, 1.48)	0.028
			MVMR Egger	1.24 (1.03, 1.49)	0.026
	ALM		MV-IVW	1.09 (1.02, 1.17)	0.011
			MVMR Egger	1.04 (0.90, 1.21)	0.60
CHD	Grip strength	Insulin resistance (fasting insulin)	MV-IVW	1.33 (1.08, 1.64)	0.008
			MVMR Egger	1.36 (1.10, 1.68)	0.004
	ALM		MV-IVW	1.17 (1.10, 1.24)	6.92×10^{-8}
			MVMR Egger	1.30 (1.14, 1.48)	9.29×10^{-5}
MI	Grip strength	Insulin resistance (fasting insulin)	MV-IVW	1.36 (1.07, 1.71)	0.011
			MVMR Egger	1.38 (1.09, 1.74)	0.008
	ALM		MV-IVW	1.15 (1.08, 1.22)	5.74×10^{-6}
			MVMR Egger	1.29 (1.12, 1.48)	3.16×10^{-4}

[a] ORs (95% CIs) represent risks for cardiometabolic diseases associated with each 1-SD lower grip strength, ALM, and WBLM.

Abbreviations: ALM, appendicular lean mass; CHD, coronary heart disease; CI, confidence interval; MI, myocardial infarction; MV-IVW, multivariable inverse-variance weighted; MVMR, multivariable Mendelian randomization; MVMR Egger, multivariable Mendelian randomization Egger; NAFLD, non-alcoholic fatty liver disease; OR, odds ratio; SD, standard deviation; WBLM, whole-body lean mass.
